# Biometric verification using oral acoustic reflections

**DOI:** 10.1371/journal.pone.0359160

**Published:** 2026-09-25

**Authors:** Amit Kam, Nir Iakoby, Daniel Tubiana, Eran Hochstadter, Roi Ram, Yaniv Zigel

**Affiliations:** 1 Echo-Id Ltd, Tel-Aviv-Yafo, Israel; 2 Biomedical Engineering Department, Ben-Gurion University of the Negev, Beer-Sheva, Israel; Fundación Universitaria del Área Andina, COLOMBIA

## Abstract

Biometric verification methods, such as voice, face, iris and fingerprint verification, are commonly used in smartphones, security systems, and medical devices. However, each involves tradeoffs in cost, accuracy, latency and susceptibility to spoofing. We present and report laboratory testing of Oral Cavity Acoustic Verification (OCAV), a biometric verification system that transmits sound waves into the user’s oral cavity, characterizes the acoustic response, and returns a verification decision by comparing the acoustic response to the individual’s prerecorded acoustic signature. A total of 108 healthy volunteers aged 18–60 years were recruited, and a unique acoustic profile was created for each participant. Across three recording sessions conducted one week apart, 20,860 genuine trials and 18,880 impostor trials were collected to evaluate verification accuracy. Verification accuracy was 90.1% for a single trial and 95.2% when pooling 5 consecutive trials. Unlike voice biometrics that depend on speech production and are sensitive to acoustic noise and audio playback attacks, OCAV exploits an active acoustic response from the oral cavity that may be less vulnerable to spoofing attacks. In contrast to other audio-based systems such as ear-canal acoustic biometrics, OCAV is intended for devices that already involve oral cavity contact. The potential applications include inhaler-based health monitoring, breath-alcohol tests, and the enforcement of nicotine-use restrictions. These capabilities can help support public health and safety efforts; for example, by monitoring youth access to e-vaping or detecting drivers’ alcohol consumption prior to ignition.

## Introduction

Biometric technologies such as voice, face, iris, and fingerprint recognition have become integral parts of modern access control and security systems [1,2].

### Related literature

Current biometric technologies involve tradeoffs between processing speed, hardware requirements, accuracy and vulnerability to spoofing. Voice recognition, for example, is highly susceptible to spoofing [3] and environmental noise [4]. Longer speech samples typically trade off speed for accuracy: studies show that the median equal-error rate (EER) drops from 23% in 2-second samples to 3% in 10-second samples [5]. Fingerprint recognition, which is known for its high accuracy, nevertheless requires specialized hardware and depends on fingerprint quality [6]. Accuracy rates range from approximately 98–99% when using advanced scanners and algorithms to 85–90% with small area capacitive sensors and simpler algorithms. Facial recognition achieves  ~ 99% accuracy in controlled tests but only 92–96% on unconstrained benchmarks when these include occlusions, pose changes, and varied lighting [7]. These systems also require high-resolution cameras and substantial computational resources, which can increase verification latency [8]. Controlled iris image-capture recognition is one of the highest-performing biometric modalities. In the NIST IREX IX evaluation, the most accurate one-to-one iris matcher achieved an estimated false-rejection rate of 0.57% at a false-acceptance rate of 0.001% [9]. However, iris recognition typically requires iris-specific imaging hardware, often near-infrared illumination, and cooperative eye positioning. Recent ear-canal acoustic methods report about 95–98% accuracy with 1–2% EER under the authors’ test conditions [10,11], thus highlighting the potential of acoustic biometrics for user verification. Table 1 summarizes different biometric approaches and highlights their limitations.

**Table 1:** Summary of related biometric approaches.

Biometric approach	General methodology	Reported performance/context	Requirements and limitations
Voice	Uses speech signals produced via the vocal tract and speaker-specific articulation patterns	EER decreases with longer speech samples, from approximately 23% in 2-second samples to 3% in 10-second samples [5]	Requires speech production, is sensitive to environmental noise and channel mismatch, and is vulnerable to replay/spoofing attacks [3,4]
Fingerprint	Uses ridge-pattern images captured by a contact sensor	High accuracy with advanced sensors (98–99%), but lower performance with small-area capacitive sensors (85–90%)	Requires specialized contact hardware and depends on sensor quality and clear ridge detail [6]
Face	Uses camera-based anatomical image capture and image-matching algorithms	Approximately 99% accuracy in controlled tests, decreasing to about 92–96% on unconstrained benchmarks that include occlusion, pose changes, and varied lighting [7]	Requires unobstructed imaging, suitable lighting, and camera hardware
Iris	Uses iris-pattern image capture, typically with iris-specific imaging hardware and near-infrared illumination	Very high verification performance under controlled iris-image capture; NIST IREX IX reported an estimated false-rejection rate of 0.57% at a false-acceptance rate of 0.001% for the most accurate one-to-one iris matcher [9]	Requires cooperative eye positioning, sufficient iris focus and resolution, limited occlusion from eyelids/eyelashes/reflections, and iris-specific imaging hardware
Ear-canal acoustic	Emits an acoustic signal into the ear canal and analyzes the reflected response	Recent acoustic ear-canal systems report approximately 95–98% accuracy and 1–2% EER under the authors’ test conditions [10,11]	Requires ear-canal access and is not naturally aligned with oral substance-exchange devices

Two recent surveys of biometrics for IoT devices identified ultra-low-power operation, protection of the biometric signature templates, and resilience to spoofing [12,13] as the three most persistent challenges and also underscored that current solutions tend to be based on cameras or fingerprint sensors that result in additional hardware overhead and on-device processing latency.

Aside from their utilization in security and access control, applications of biometric technologies are increasingly being explored for healthcare and substance-use monitoring. A recent systematic review of real-time patient identification evaluated fingerprint, face, and iris systems on accuracy, response time, usability, privacy, hygiene, safety, and operational factors [14]. An expert commentary argued that biometrics can reduce identification errors and improve safety, while highlighting infrastructure, privacy, and patient acceptance as key implementation constraints [15]. Other potential uses include preventing underage misuse of electronic cigarettes or vapes by identifying the designated user [16]. In cars that have a breathalyzer to test the alcohol level of the driver before ignition, biometric devices can confirm that the person taking the test is the actual driver [17]. These widely different uses demonstrate the versatility of biometric technologies beyond traditional security roles and confirm their relevance to public health and safety.

### Study objective and main contribution

**Fig 1:**
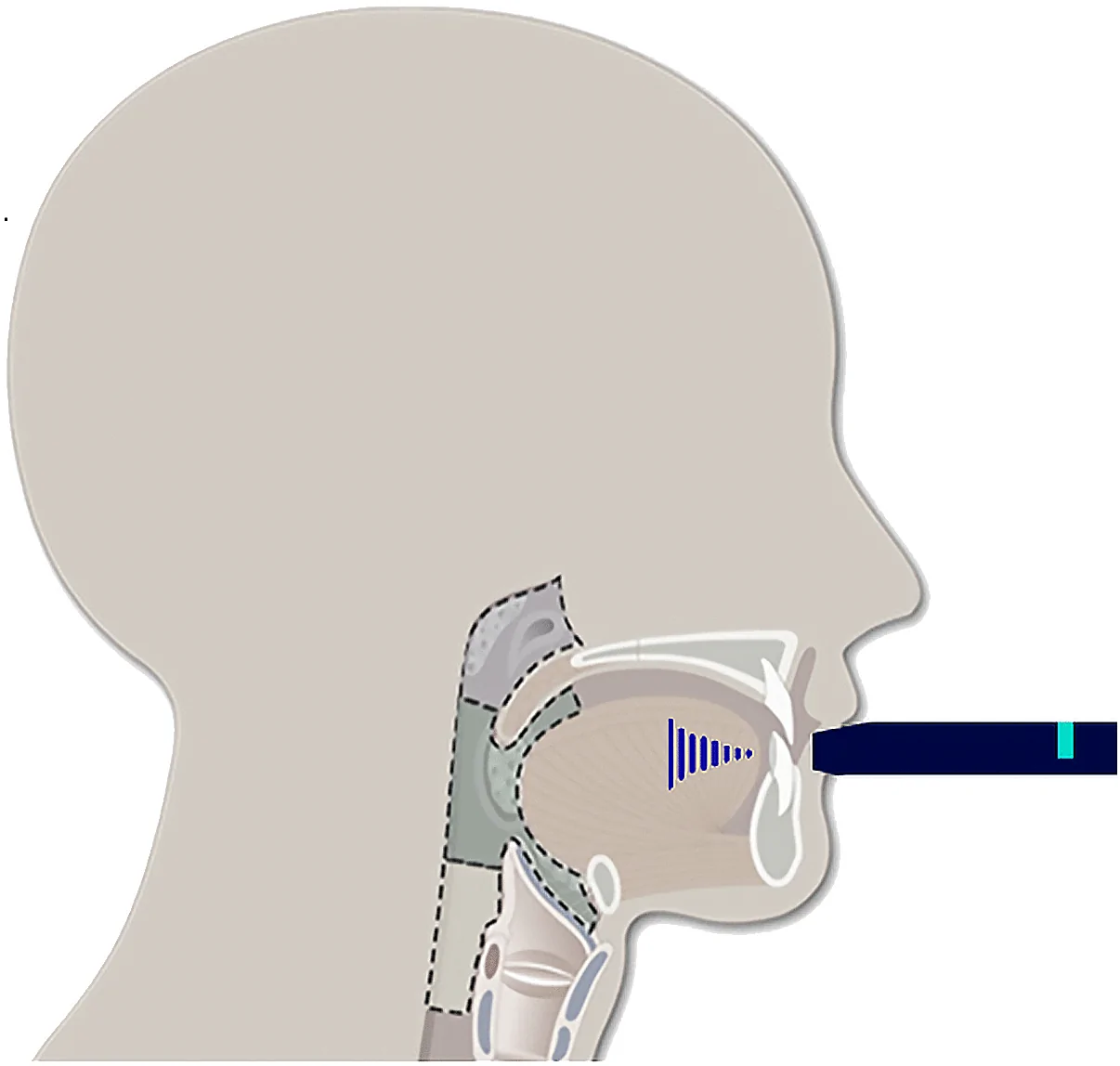
The oral cavity acoustics verification system concept integrated into an electronic cigarette.

The main goal of this study was to develop and evaluate a low-cost, oral-acoustic biometric verification system that delivers sub-second, spoof-resistant user verification on resource-constrained devices. This article describes the Oral Cavity Acoustic Verification system (OCAV), a new biometric approach and device that capitalizes on oral cavity acoustic reflections generated during natural user inhalation to securely identify users. To the best of our knowledge, oral cavity acoustic reflections have never been implemented in biometric verification. By embedding authentication in the user’s natural inhalation behavior, the OCAV system enhances security with minimal user effort. The technology can be applied to multiple cases, including with important public health and safety applications such as eliminating spoof identification in electronic nicotine delivery systems (“vapes”), preventing breathalyzer spoofing, and validating patient identity in medical inhalers. By aligning biometric verification with habitual user behaviors at the point of oral substance exchange, the OCAV combines user-facing identification with a secure authentication method. Since verification takes place during inhalation, the OCAV system ensures that the authenticated individual is the one inhaling. For example, integrating this technology into e‑cigarettes could prevent unauthorized, including underage, use of e-cigarettes, by conducting an identity verification test at each inhalation (Fig 1).

This feasibility study makes three main contributions:

- Presents an oral acoustic verification approach that uses inhalation-triggered acoustic reflections from the oral cavity for user verification.
- Describes the experimental workflow, including profile enrollment, transfer-function estimation, feature extraction, and verification scoring.
- Evaluates the approach on a multi-session cohort of adult participants and reports genuine/impostor verification performance, including accuracy, AUC, EER, and subgroup analyses.

## Materials and methods

### Study design

A prospective study was conducted from December 2023 to April 2024 at the Biomedical Signal Processing Research Laboratory, Ben-Gurion University of the Negev, Israel, to evaluate the performance of the OCAV system.

#### Sampling and participants.

A total of 108 healthy adult participants were recruited for this study between December 17, 2023 and April 15, 2024, primarily from postings at Ben Gurion University of the Negev. They ranged in age from 18 to 60 and were evenly stratified into three age groups (18–30, 31–43, and 44–60), with 36 participants in each group.

#### Ethics.

Each participant was provided with detailed information about the trial, its purpose, procedures, and potential risks, and signed an informed consent form prior to participation. To protect participant confidentiality, participant data were key-coded using serial numbers (e.g., A001_171223). The study protocol was approved by the Ben‑Gurion University Human Subjects Research Committee (approval no. 467‑3). All procedures adhered to the Committee’s guidelines. Written informed consent was obtained from each participant before enrollment.

**Safety and compliance:** Safety measures were stringently enforced throughout the study to safeguard the well-being of all participants. Although there were no imminent risks to the participants in any form, at the end of each session, rigorous sterilization procedures were undertaken for each mouthpiece, accompanied by appropriate labeling for accurate identification of the respective participant’s mouthpiece allocation. The paramount concern throughout the study was stringent adherence to safety protocols and regulatory standards.

#### Eligibility criteria and demographics.

Candidates were excluded if pregnant, had any type of facial malformation that prevented full closure of the mouth on the mouthpiece or surgery or procedures affecting mouth structure. Table 2 lists the participants’ age, BMI, gender, and smoking status by age group.

**Table 2:** Demographics by age group (μ±σ).

	Ages 18–30	Ages 31–43	Ages 44–60
Age (years)	25.6±1.7	36.1±3.9	50.7±5
BMI (kg/m^2^)	23±2.9	24.9±4.3	26.4±4.6
No. male (female) subjects	12 (24)	9 (27)	7 (29)
No. smokers (non-smokers)	16 (20)	15 (21)	14 (22)

### The inhalation device

**Fig 2:**
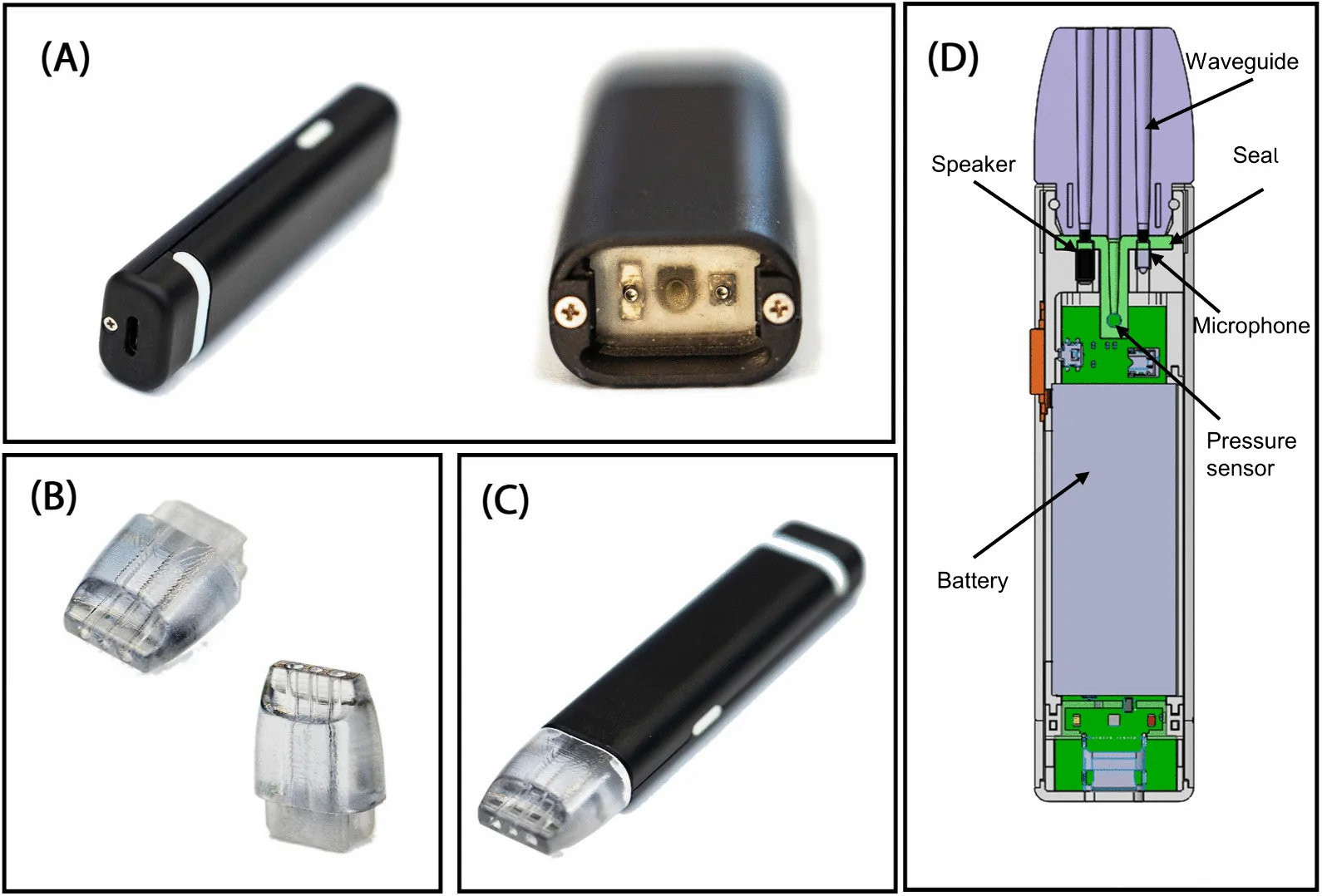
The oral cavity acoustic verification device: front, side and top views. (A) Device without mouthpiece, (B) Mouthpiece, (C) Device assembled with mouthpiece, (D) Schematic diagram labelling main components.

The OCAV device resembles an e-cigarette (Fig 2) and measures 120 mm × 20 mm × 15 mm (weight ≈ 35 g). It is pharmacologically inert and contains no nicotine, propylene glycol, vegetable glycerin, or flavoring additives commonly found in commercial e-liquids. It is powered by a rechargeable battery (connected via USB Type-C) and has a removable mouthpiece. The mouthpiece is 3D printed from biocompatible materials, ensuring safety during contact with bodily tissues. During the study, each participant was assigned a personal, dedicated mouthpiece that was sterilized between visits and discarded after the final session. The device is activated when the participant inhales. The inhalation-induced drop in internal pressure activates the pressure sensor, which then prompts the speakerphone to emit a 50 ms chirp (2–8 kHz) acoustic signal. Simultaneously, the on-board microphone captures the acoustic echoes from the participant’s oral cavity and digitizes them at 44.1 kHz (16 bit/sample) before streaming to a laptop for analysis.

The OCAV system maps the acoustic transfer function (frequency-response curve) of the individual’s oral cavity by analyzing inhalation-triggered echo signals. Because each person’s oral-cavity geometry is different, it produces a characteristic acoustic transfer function. To verify identity, each new recording is compared to the participant’s stored profile generated during registration.

#### Feature extraction.

**Fig 3:**
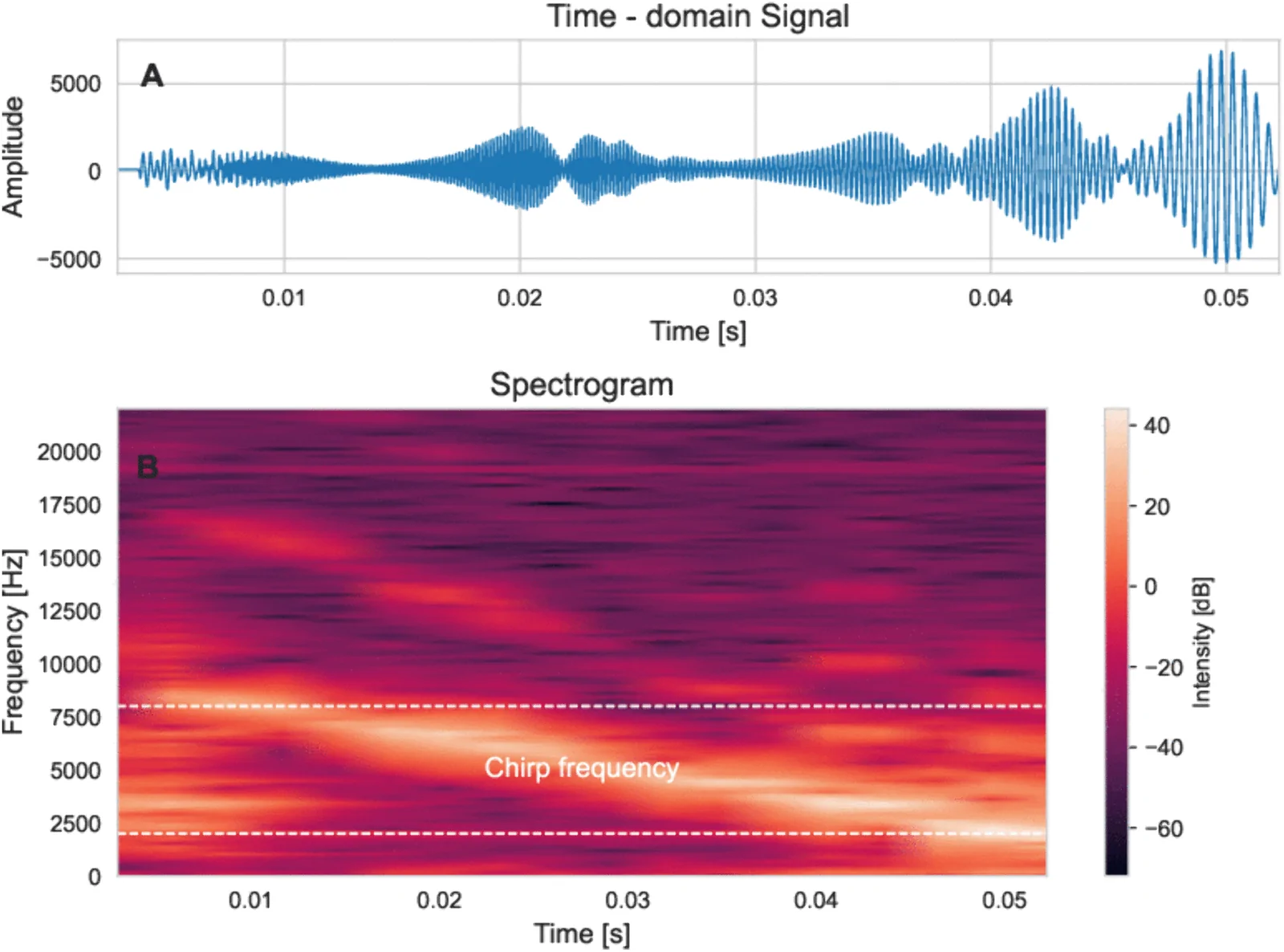
Typical oral acoustic reflection signal. (A) time-domain waveform and (B) spectrogram.

An oral acoustic reflection signal is recorded from each inhalation, y(n). Fig 3 illustrates a typical acoustic reflection, displayed as both a time-domain waveform and its spectrogram.

**Fig 4:**
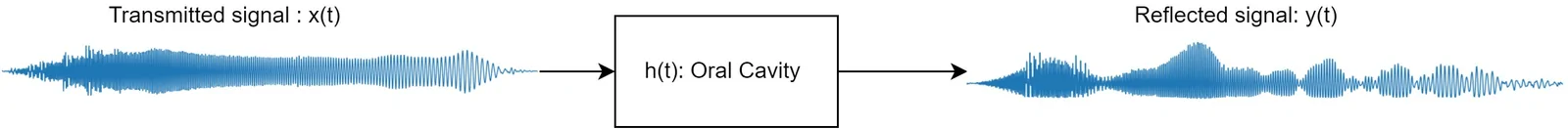
Oral cavity modulation.

The oral cavity is modeled as a linear-time-invariant (LTI) acoustic system with frequency response *H*(*jω*), as described in previous vocal-tract acoustics studies [18,19]. Because the transmitted signal was known and the oral reflected signal was captured, the transfer function can be estimated by dividing the Fourier spectra (4096-point FFT, Hann window) of the reflected signal, Y(jω)=F{y(n)}, and the transmitted signal, X(jω)=F{x(n)}, as shown in Eq. (1) and illustrated in Fig 4.


$$\begin{array}{c} \hat{H}(j\omega )=\frac{Y(j\omega )}{X(j\omega )} \end{array}$$ (1)

**Fig 5:**
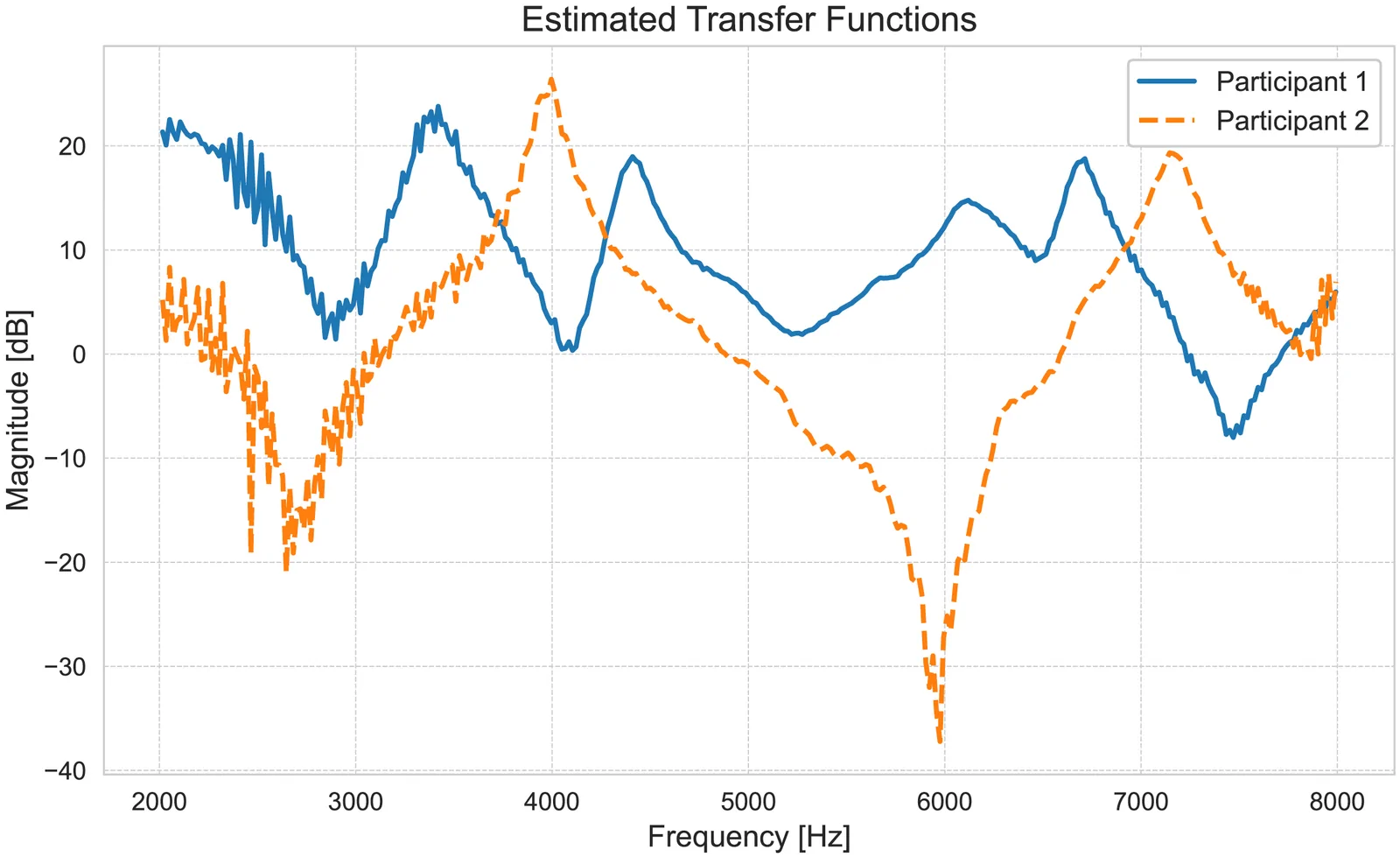
Estimated Transfer functions of two different participants.

Fig 5 compares the magnitude of the estimated transfer functions of two representative participants to illustrate the inter-subject variability exploited by the verification model.

The estimated transfer function was also used to reconstruct the components of the recording explained by the oral acoustic response. As defined in Eq. 2, when y(n) is the recorded signal, x(n) is the transmitted chirp and $\hat{h}(n)=F^{-\text{1}}\left\{\hat{H}(j\omega )\right\}$ is the impulse response corresponding to the estimated transfer function, the reconstructed response $\hat{y}(n)$ and recording residual r(n) is as follows:


$$\begin{array}{cc} \hat{y}(n) & =x(n)*\hat{h}(n)\\ r(n) & =y(n)-\hat{y}(n) \end{array}$$ (2)

A 342-dimensional descriptive feature vector is then extracted from each estimated transfer function by concatenating the predefined descriptor groups into a single representation. This feature set summarizes the acoustic response in terms of the spectral magnitude patterns across the analyzed frequency range, the spectral structure of the early reflection component, the frequency-banded recording-residual characteristics, and permitted the extraction of global signal-quality measures such as clean-signal power, recording-noise power, and the SNR to capture the stable shape of the oral acoustic response and recording quality information. Dimensionality reduction was not applied in the current study before classification to preserve the direct interpretability of the predefined acoustic-response and signal-quality descriptors.

#### Enrollment process: Personal profile formation.

**Fig 6:**
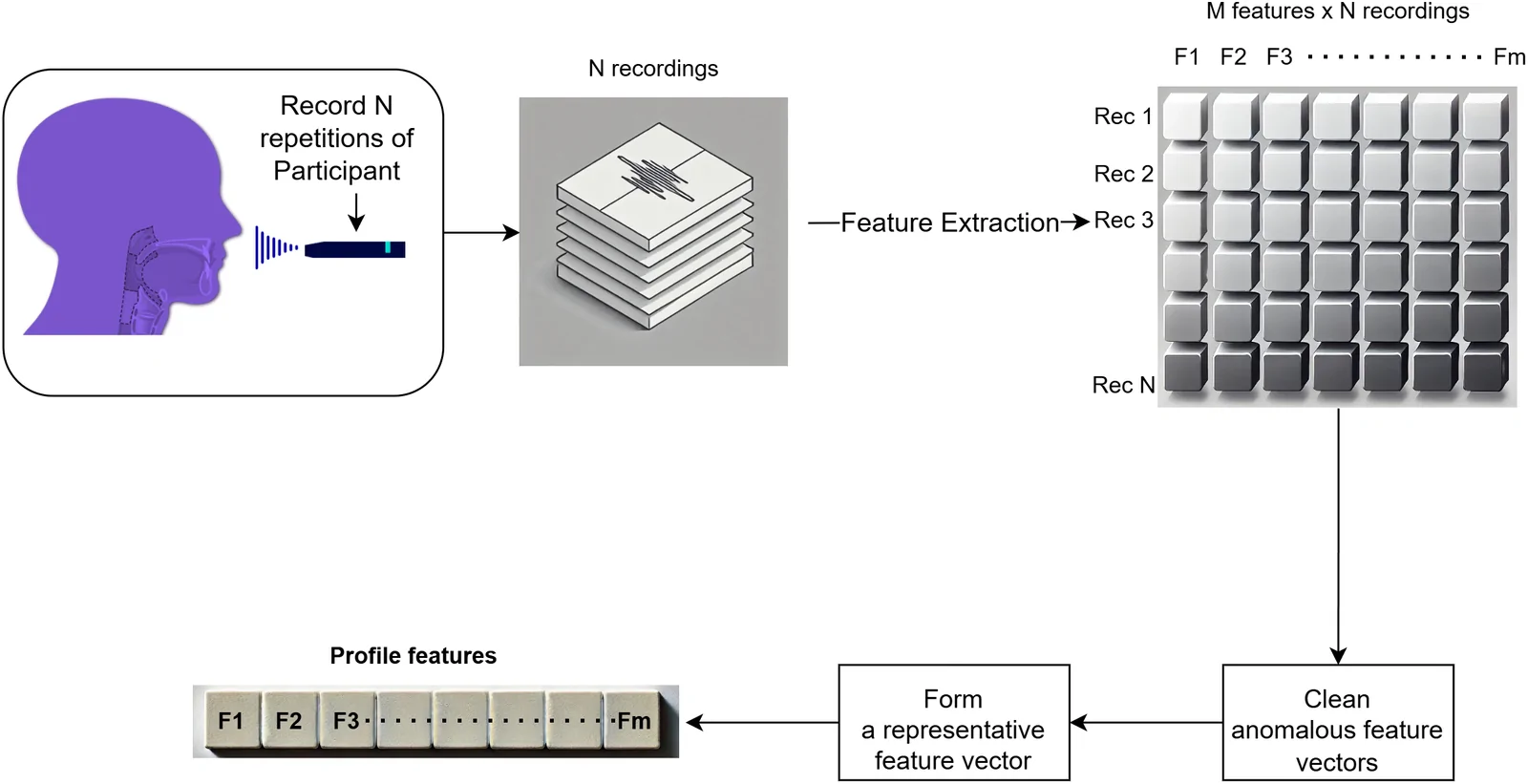
Registration workflow from inhalation recordings to personal profile creation.

During user registration, 20 consecutive inhalation-triggered recordings were obtained from each participant. These recordings were processed as illustrated in Fig 6. For each recording, a descriptive feature vector was extracted. Outlier feature vectors identified by an Isolation Forest model (200 trees, contamination≈4%) were discarded. Specifically, the Isolation Forest was applied to recording quality features rather than to the final verification classifier output. These quality features included clean signal variance, pairwise consistency measures, correlation, smoothed spectrum deviation, skewness, kurtosis, and spectral flatness. This unsupervised step was used to remove atypical recordings before profile formation. The remaining vectors were averaged elementwise to yield a single representative vector, which was stored as the participant’s profile template.

#### Verification process: Verifying claim of identity.

**Fig 7:**
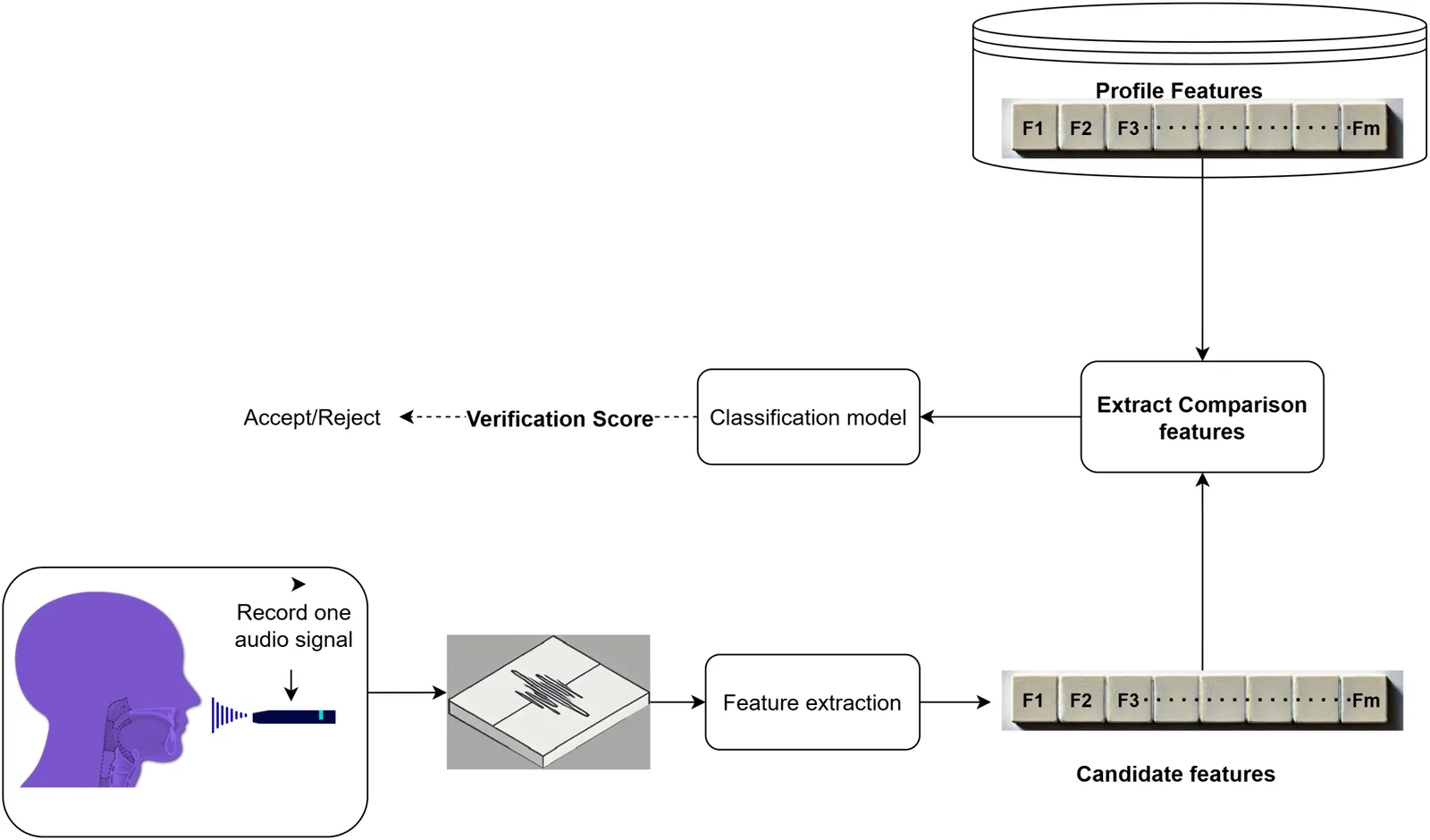
Verification workflow.

Upon an identity claim, a single inhalation-triggered recording is captured with the OCAV device. A 342-dimensional feature vector from each verification recording is extracted, using the same spectral energy and bandwidth features as described for profile creation. A comparison feature vector is constructed by pairing the candidate and template vectors and computing similarity-based descriptors. The resulting 242-dimensional comparison feature vector captures the pairwise relationship by concatenating absolute element-wise differences, signed differences, and cosine-normalized distances between the candidate and template features. The comparison vector is fed into a LightGBM gradient-boosted decision-tree classifier (200 trees, learning rate = 0.1), which returns a scalar verification score in the 0–1 range. Fig 7 illustrates the verification workflow, from single inhalation through feature extraction, comparison-vector construction, and LightGBM score output.

### Classification model

To verify user identity, a Light Gradient Boosting Machine (LightGBM) machine-learning classifier [20] optimized for distributed learning and memory efficiency was implemented [21]. Because LightGBM grows trees leaf-wise by selecting splits that maximize loss reduction, it often outperforms level-wise algorithms such as XGBoost, as shown in finance-sector benchmarks (RMSE = 0.76 vs 0.83) [22].

The comparison between the candidate and profile transfer-function spectra is fed into the LightGBM classifier. For the current study, the LightGBM classifier was trained on a pre-existing in-house dataset of 20,000 comparison vectors (10,000 genuine and 10,000 impostor trials) thereby ensuring a 1:1 class balance. The participants enrolled to generate this training dataset were not included in the 108-participant evaluation cohort reported here. Model development and parameter selection were performed before evaluation and the data from the present study cohort was not used for classifier training to reduce the risk of overfitting to the present cohort.

The LightGBM classifier outputs a verification score on a continuous scale 0 ≤ s ≤ 1, where higher values indicate greater similarity between the recording and the claimed profile. The system compares the verification score to a fixed threshold (τ), where scores ≥ τ are considered as genuine (positive), and otherwise rejected (negative). A positive result is declared when the identity claim is accepted, and a negative result is declared when the claim is rejected. The classifier considers an identity claim to be *genuine* when it matches the verification recording to the participant’s own profile. A match to any other participant’s profile constitutes a false identity claim, and constitutes an *impostor* trial.

### Experimental protocol

**Fig 8:**
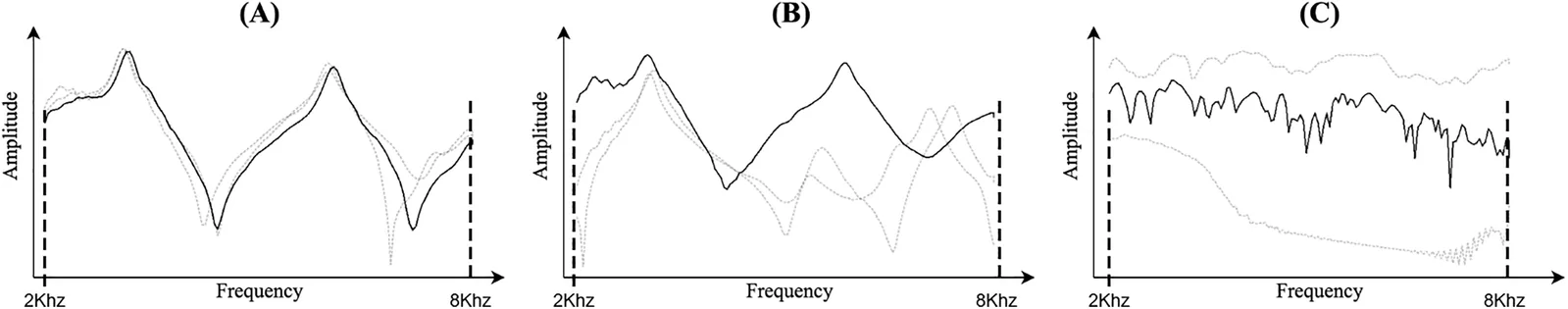
Acoustic echo waveforms captured during user inhalations. The dark line represents the current inhalation, and the lighter line shows the immediately preceding inhalations. (A) Consistent inhalations, (B) Inconsistent inhalations, (C) Noisy signals.

We hypothesized that each participant’s transfer function would be sufficiently distinctive to support identity verification. The experiment was conducted in a quiet office environment. Each participant underwent three consecutive sessions at intervals of approximately one week. During each session, participants took brief inhalations from the device. Participants first completed a structured pre-training session during which proper device use was demonstrated and practiced. The main objective was to instruct the participants to breathe in a uniform manner so that the resulting transfer-function plots would be comparable across trials. The participants were also taught to make sure that the mouthpiece was firmly connected to the device to maintain an airtight seal. This airtight seal also helps prevent background noise from interfering with the received signal. Training began with a provisional profile. Once verified, participants recorded the full 20-inhalation profile (Session 1 only). The testing step collected 80 inhalations. Fig 8 shows example signals obtained from participants’ inhalations using the device.

**Fig 9:**
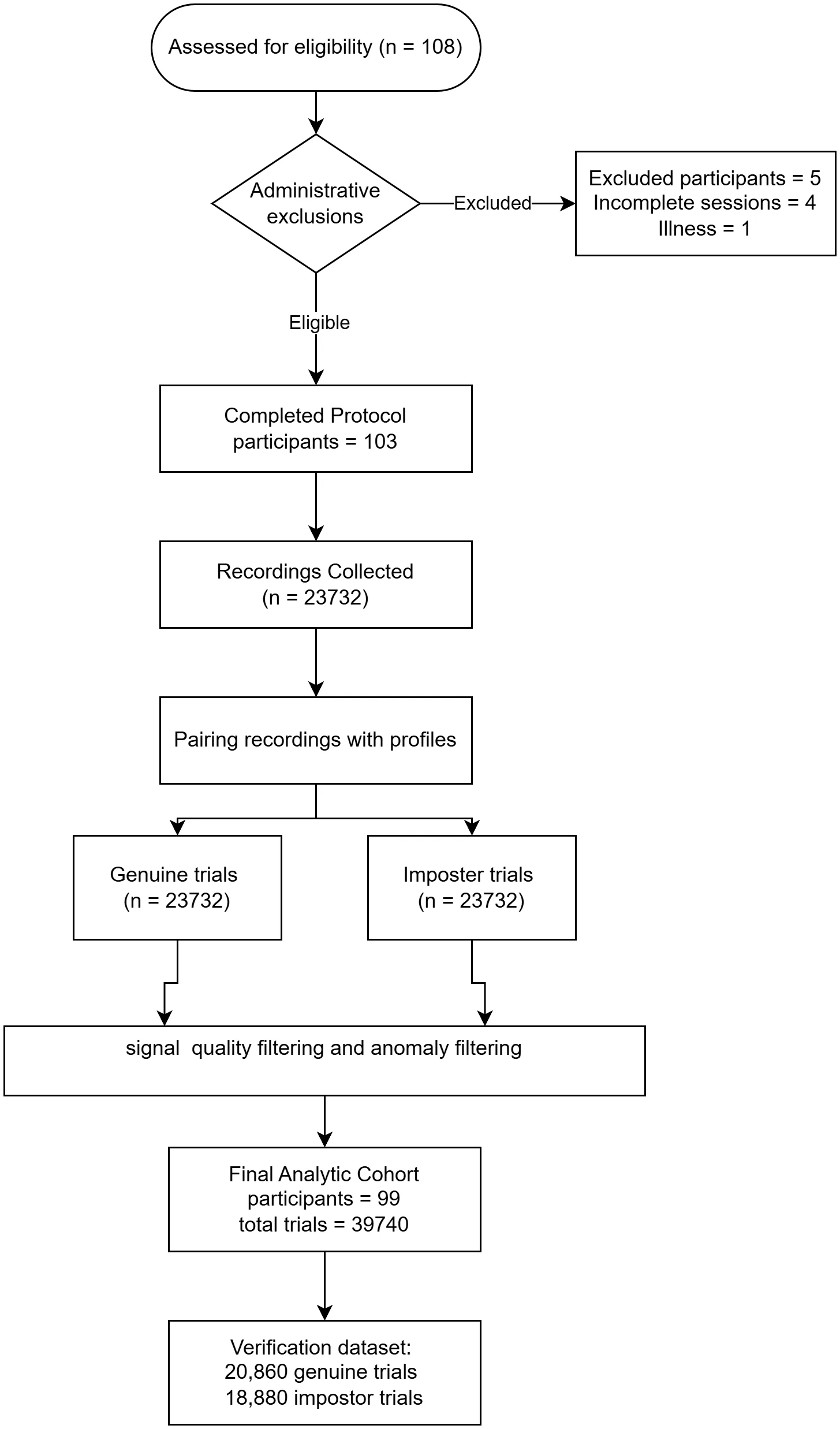
Data flow.

Fig 9 shows the data flow from enrollment to analysis. Of the 108 participants, 5 were excluded, leaving 103 profiles (2,060 enrollment recordings) and 23,732 session recordings. Each session recording was paired with its owner’s profile (genuine) and with an impostor profile sampled at random (seed = 42), yielding 47,464 trials. After quality control (variance gate and unsupervised outlier filtering), 39,740 trials remained (20,860 genuine; 18,880 impostor). See S1 Appendix for step counts and rationales.

#### Performance metrics & statistical analyses.

The primary endpoints for assessing system performance were the overall single-trial accuracy and the area under the receiver operating characteristic (ROC) curve. These endpoints were selected to provide both application-level and threshold-independent performance measures. The secondary metrics included the equal error rate (EER) as well as the precision, recall, and F1 scores. The confidence intervals for these metrics and the ROC curves were computed using bias-corrected bootstrap methods (1,000 samples). Data normality was examined using the Shapiro-Wilk and Kolmogorov-Smirnov tests (significance threshold p < 0.001), which confirmed non-normal distributions. For the two-group comparisons (smokers vs. non-smokers), Mann-Whitney U tests with Bonferroni correction for multiple comparisons were applied. For analyses involving three or more unrelated groups (e.g., age brackets), a Kruskal-Wallis test followed by a Dunn-Bonferroni post hoc correction was utilized. Friedman tests with Nemenyi post hoc analyses were employed to examine repeated-measure effects (across visits). All analyses were conducted using Python 3.9 and associated libraries (NumPy 1.26 [23], Pandas 2.2 [24], SciPy 1.12 [25], scikit-learn 1.5 [26], matplotlib 3.9 [27], seaborn 0.13 [28]). All analyses were done on a standard desktop workstation (Intel Core i7 CPU, 32 GB RAM, no GPU acceleration was required).

## Results

**Fig 10:**
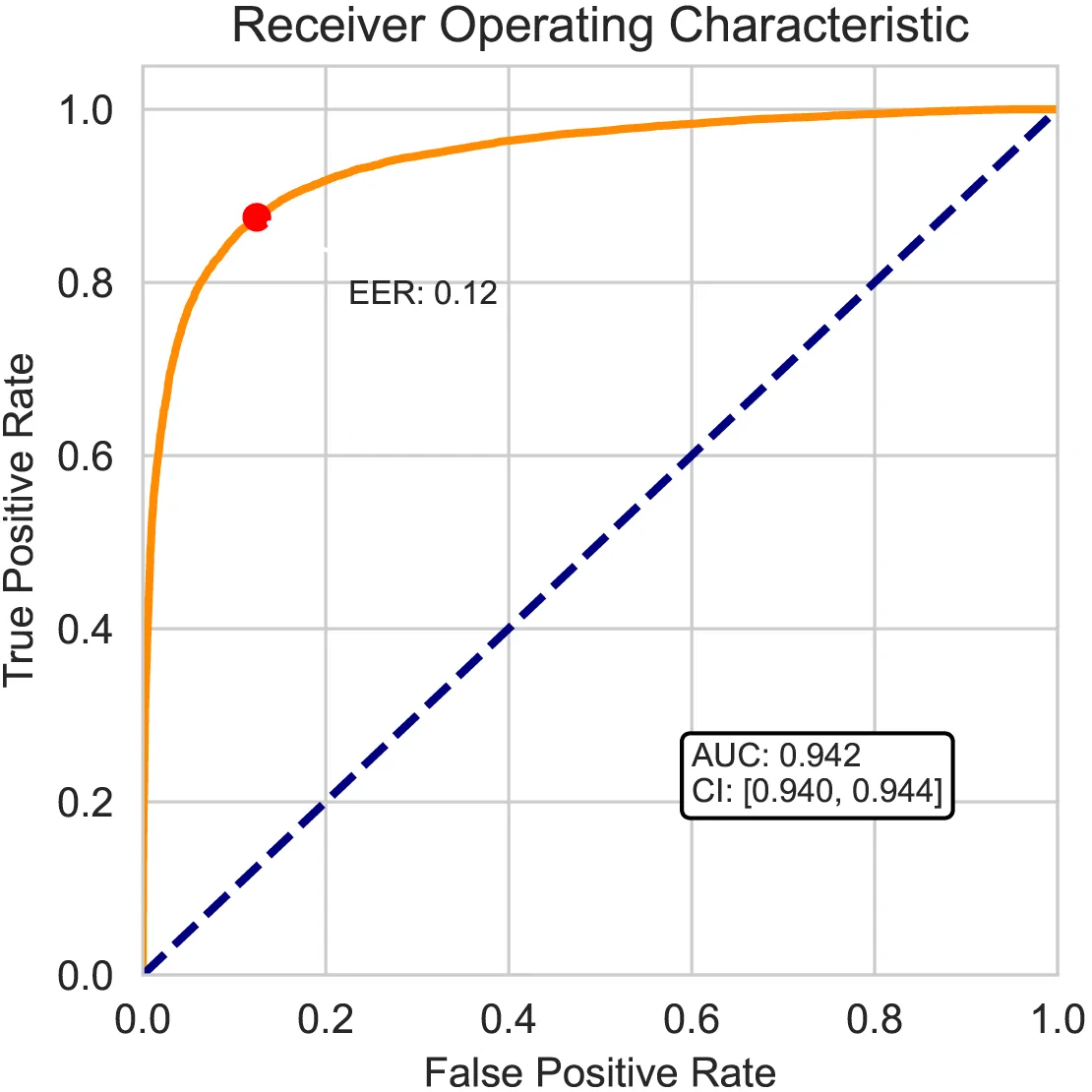
ROC curve plot of all data. The ROC curve illustrates the tradeoff between the true positive rate (sensitivity) and false positive rate (1-specificity) for the OCAV system. Curves farther from the diagonal indicate better genuine/impostor discrimination. Based on 20,860 genuine trials and 18,880 impostor trials from 99 subjects, the AUC was 0.942 (95% CI: 0.940–0.944).

**Fig 11:**
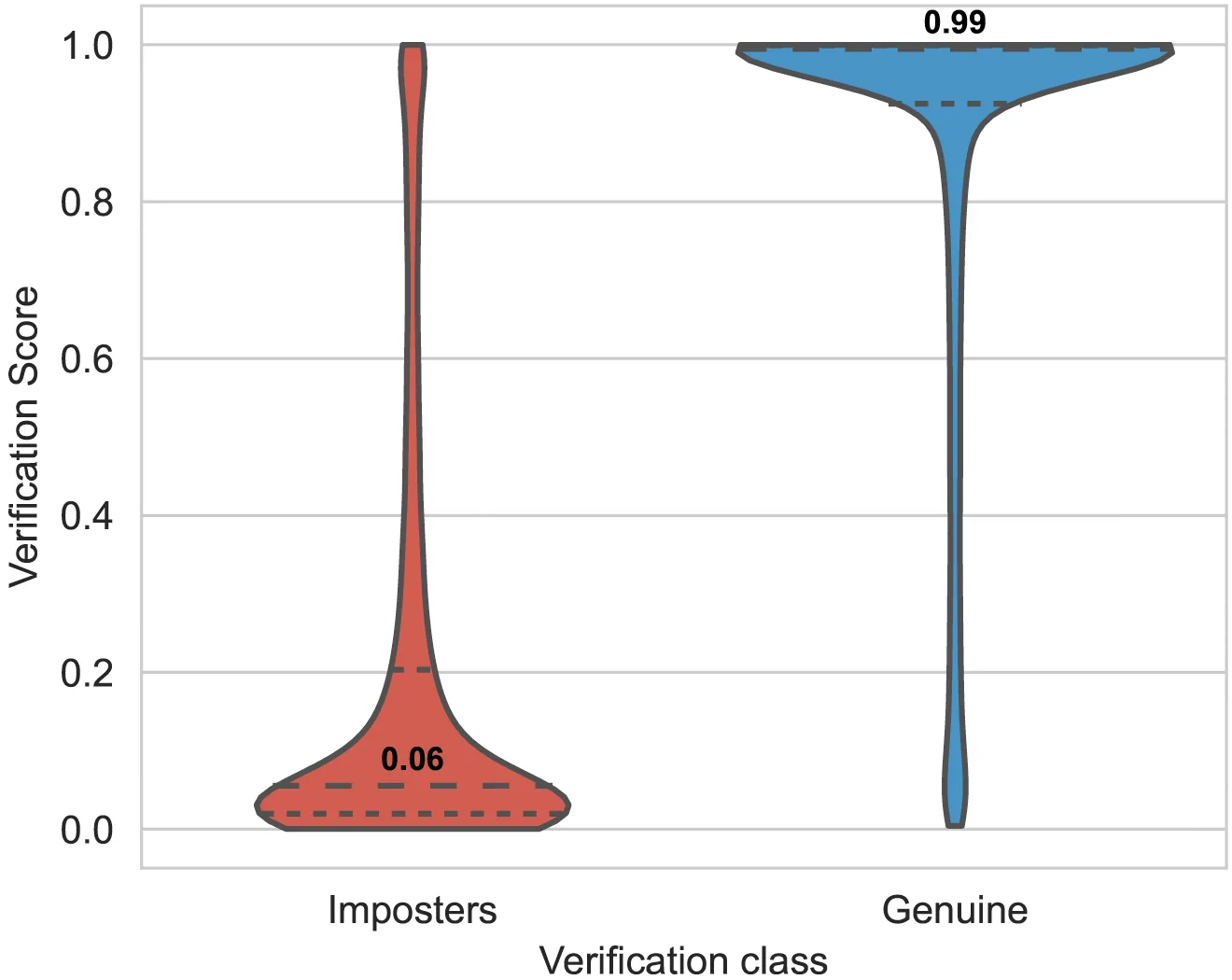
Distribution of verification confidence scores for genuine and impostor trials. Violin plots depicting the distribution of verification confidence scores. The width of each violin represents the probability density of the data. The central line indicates the median score (value shown), and the inner lines denote the interquartile range. A greater separation between genuine and impostor score distributions indicates better verification discrimination.

The results focused on verifying whether a claimed identity matched the enrolled profile. We report the EER, the operating point where the false-acceptance and false-rejection rates coincided, and overall accuracy. The ROC curve was plotted; i.e., sensitivity (true positive rate) versus the false positive rate to evaluate system discrimination for 20,860 genuine and 18,880 imposters trials (Fig 10). The Area Under the ROC Curve (AUC) was 0.942 (95% CI, bootstrap 0.940–0.944). Fig 11 shows the distribution of the verification scores for all genuine and impostor trials. The analysis implemented two thresholding strategies. First, a global threshold was applied uniformly across all participants, which yielded an overall accuracy of 88%. Then, to explore the system's potential under optimal conditions, we determined individual thresholds for each participant, which yielded an improved accuracy of 90%. Although participant-specific thresholds can enhance performance, they may introduce overfitting and reduce generalizability. Therefore, we report accuracy based on both the global and individual threshold results to provide a comprehensive evaluation. All the analyses and tables below are based on the participant-specific thresholds to illustrate the system's best-case performance. We improved the overall verification accuracy by aggregating up to five consecutive tests, from which we selected the maximum verification score within each window. The accuracy increased from 90.1% for one test to 95.2% for five tests. Each inhalation test required approximately 150 ms to capture and process. Consequently, aggregating five consecutive tests took well under one second (~750ms), confirming that multi-sample authentication remained compatible with real-time applications. Table 3 lists all performance measures from one to five consecutive tests.

**Table 3:** Consecutive test results (%).

Consecutive tests	Accuracy (%)	Recall (%)	Precision (%)	F1 (%)	EER (%)
1	90.1	90.2	90.9	90.6	12.5
2	92.5	93.9	92.8	93.4	10.5
3	93.7	96.1	93.5	94.8	9.7
4	94.4	97.0	94.5	95.7	9.3
5	95.2	98.4	94.8	96.6	9.3

### Normality test

The normality of the verification confidence scores (n = 39 740) was assessed using Shapiro-Wilk tests (W = 0.55, p < 0.001) and Kolmogorov-Smirnov tests (D = 0.58, p < 0.001), both of which rejected the null hypothesis of a normal distribution. Consequently, non-parametric analyses were employed: Mann-Whitney U tests for two-group comparisons, Kruskal-Wallis tests with Dunn-Bonferroni post hoc corrections for multi-group (age) comparisons, and Friedman tests with Nemenyi post-hoc analyses for repeated-measure comparisons.

### Performance by visit

To assess temporal stability, the verification scores were analyzed separately for each of the three weekly sessions (Visit 1: 7,240 genuine and 6,607 impostor tests, visit 2: 6,854 genuine and 6,095 impostor tests, visit 3: 6,766 genuine and 6,178 impostor tests)

**Fig 12:**
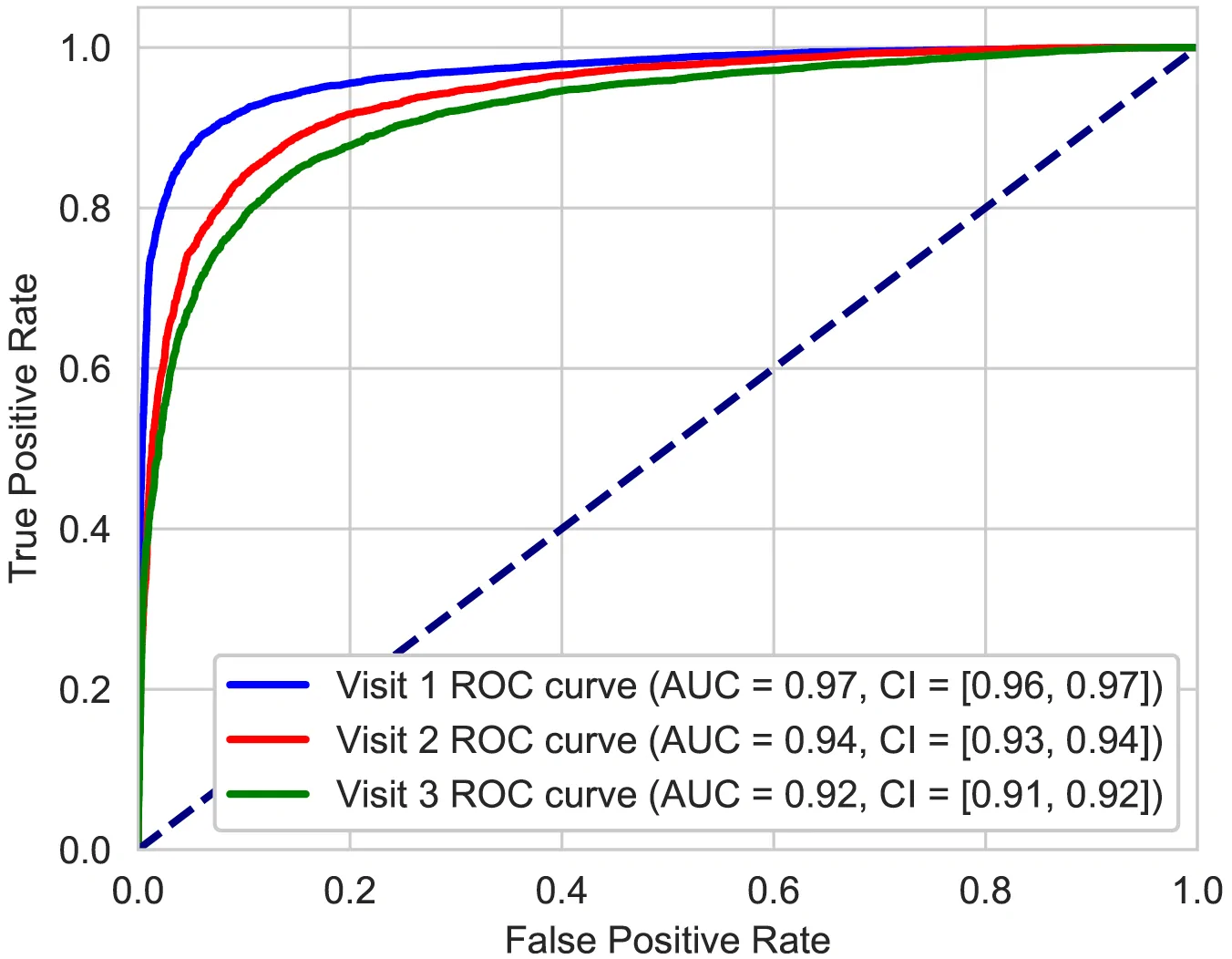
ROC plots by visits. ROC curves for Visit 1, Visit 2 and Visit 3. Each curve plots the true-positive rate (genuine-acceptance rate) against the false-positive rate (impostor-acceptance rate). Curves farther from the diagonal indicate stronger genuine/impostor discrimination. The data are based on 7,240/6,607, 6,854/6,095 and 6,766/6,178 genuine/impostor verification trials for Visits 1–3, respectively. The dashed diagonal indicates chance performance.

**Fig 13:**
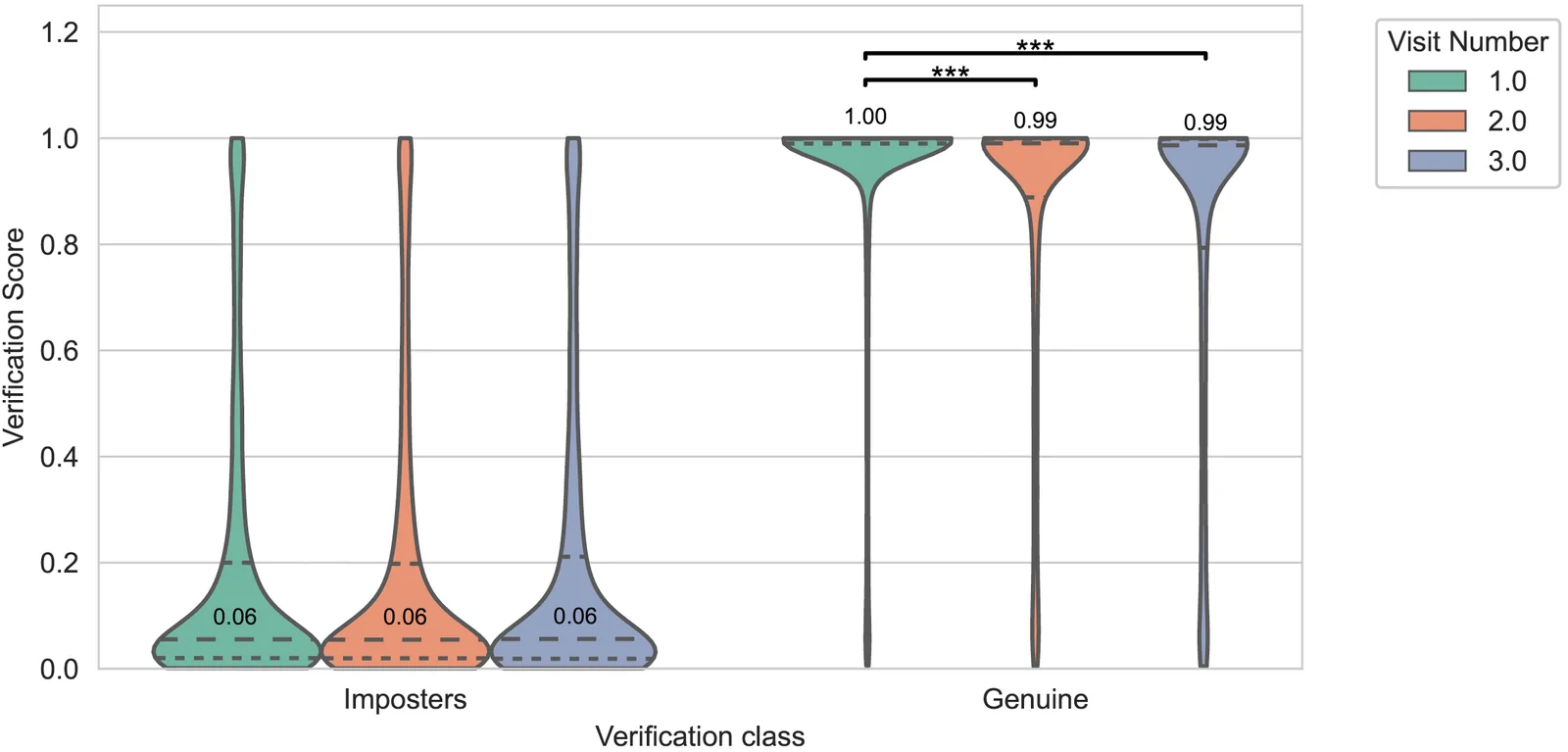
Verification-score distributions by visit. Violin plots showing the similarity-score distributions for genuine (right) and impostor (left) trials across the three weekly sessions. A greater separation between genuine and impostor score distributions indicates better verification discrimination. The central line marks the median; the two outer lines mark the inter-quartile range. Asterisks indicate pair-wise differences that remained significant after Nemenyi correction (*** p < 0.001).

Fig 12 presents the ROC curves for Visits 1–3. Fig 13 shows violin plots of the verification-score distributions for genuine and impostor trials across these visits.

The Friedman test comparing verification scores across the three visits returned a significant difference in genuine scores (χ^2^_2_ = 73.2, p < 0.001) but no difference in imposter scores (χ^2^_2_ = 0.06, p = 0.97). Thus., there was a statistically significant difference between visits in the expected true results. There was no significant difference in expected false scores. Given the significant Friedman test results for the expected true scores, pairwise comparisons were conducted using the Nemenyi test to identify which visits differed. The Nemenyi test indicated significant differences between Visit 1 and Visit 2 (p = 0.001) and between Visit 1 and Visit 3 (p = 0.001). There was no significant difference between Visit 2 and Visit 3 (p = 0.53).

### Results by age group

**Fig 14:**
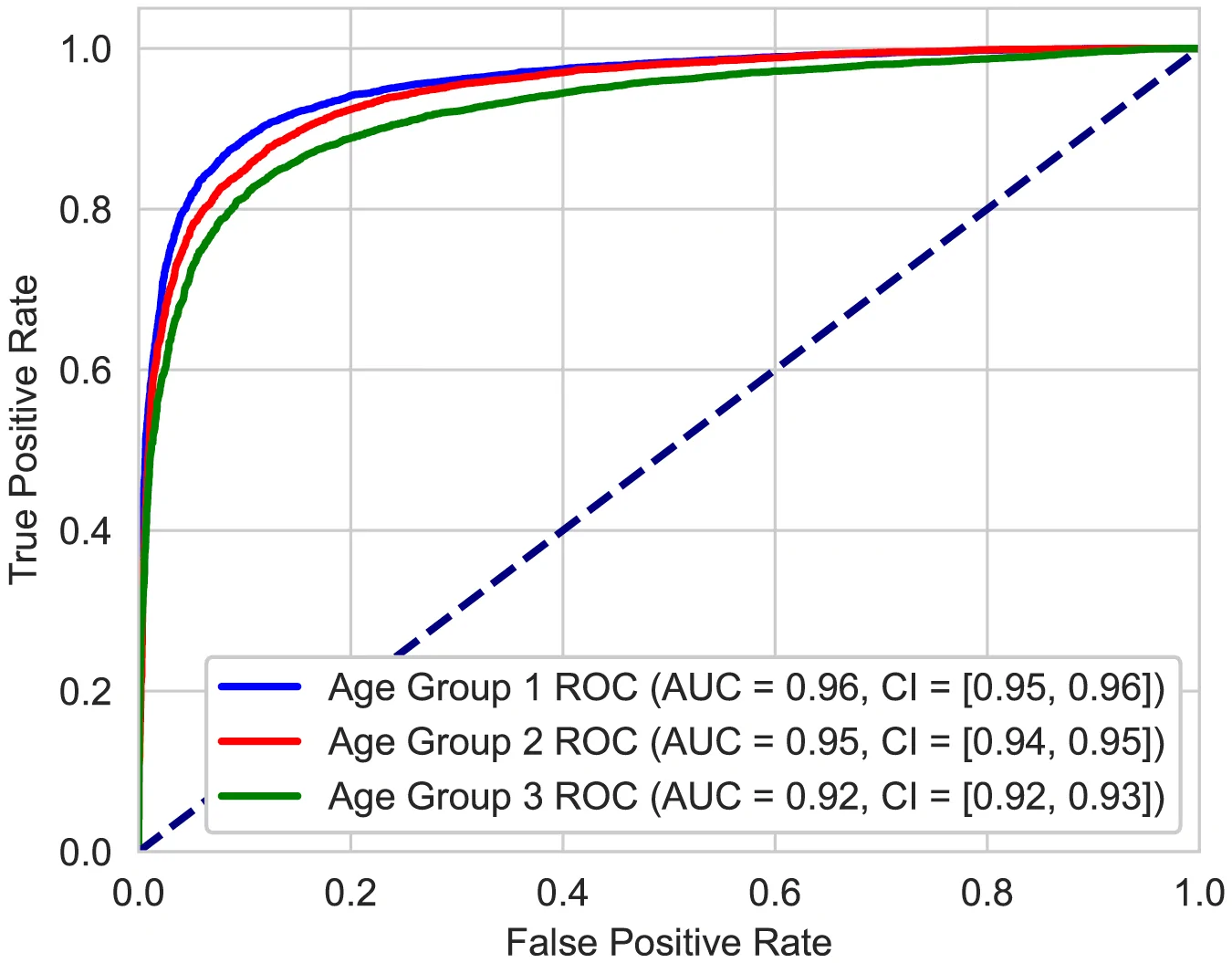
ROC curves by age groups. Receiver-operating-characteristic curves for the three age brackets: 18–30 (Group 1), 31–43 (Group 2), and 44–60 (Group 3). Each curve plots the genuine-acceptance rate against the impostor-acceptance rate, derived from 7,038/6,556 (Group 1), 6,747/6,118 (Group 2) and 7,075/6,206 (Group 3) genuine/impostor trials, respectively. Curves farther from the diagonal indicate greater genuine/impostor discrimination.

**Fig 15:**
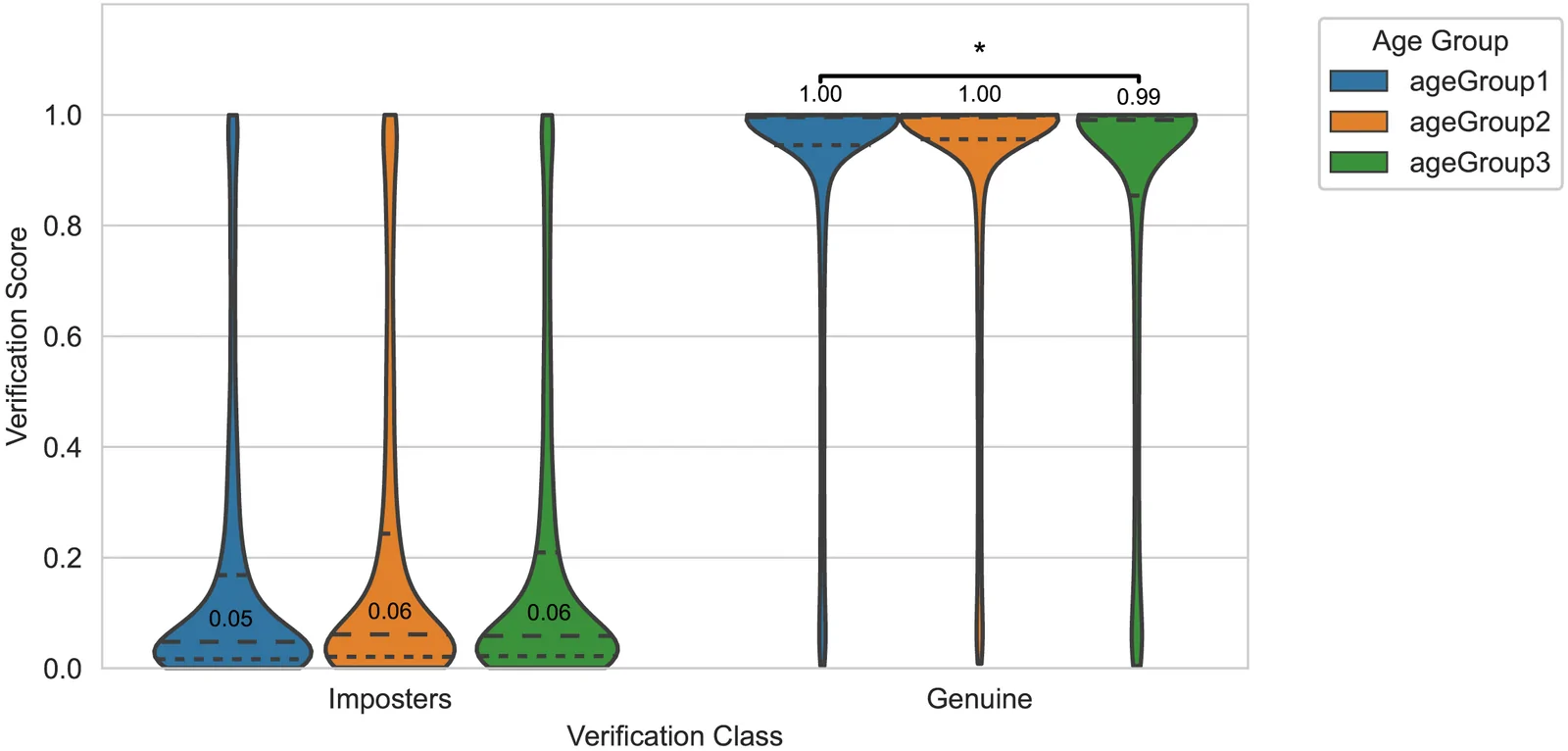
Violin plots of prediction confidence scores by True Target (False and True) and Age Group. Violin plots showing similarity-score distributions for genuine (right half of each violin) and impostor (left half) trials across the three age brackets. Each violin represents the distribution of scores for a specific age group within each verification category, genuine or imposter. A greater separation between genuine and impostor score distributions indicates better verification discrimination. Significant differences between age groups are indicated with asterisks above the dotted lines (*p < 0.05).

To compare system performance across age groups, we created three evaluation subsets. Age Group 1 contributed 7,038 genuine and 6,556 impostor trials; Age Group 2 contributed 6,747 genuine and 6,118 impostor trials; and Age Group 3 contributed 7,075 genuine and 6,206 impostor trials. Performance by age group is presented in Fig 14 as ROC curves. Fig 15 illustrates the verification score distributions across age groups, with brackets indicating statistically significant pairwise differences based on the Kruskal-Wallis and Mann-Whitney U tests.

A Kruskal-Wallis H test revealed a statistically significant difference in verification scores across the three age groups for the expected genuine trials (H = 7.5, p = 0.02). Pairwise comparisons using the Mann-Whitney U test showed a statistically significant difference between Age Group 1 and Age Group 3 after Bonferroni correction (adjusted p = 0.04 < α = 0.05). No significant differences were found between the other pairs of age groups using Mann–Whitney U tests with Bonferroni correction (all adjusted p > 0.05). For the impostor trials, the Kruskal-Wallis H test indicated a significant difference across age groups (H = 6.7, p = 0.034). However, none of the pairwise comparisons between age groups were statistically significant after applying the Bonferroni correction (smallest adjusted p-value = 0.056).

### Results by smoker/non-smoker groups

Verification metrics were also stratified by self‑reported smoking history. Smokers were defined as individuals who identified themselves as occasional, intermittent, or non-daily smokers, either currently or in the past. The type of smoked substance was not considered relevant to this analysis. The dataset comprised 8,760 genuine and 8,083 impostor trials from smokers, and 12,100 genuine and 10,797 impostor trials from non‑smokers.

**Fig 16:**
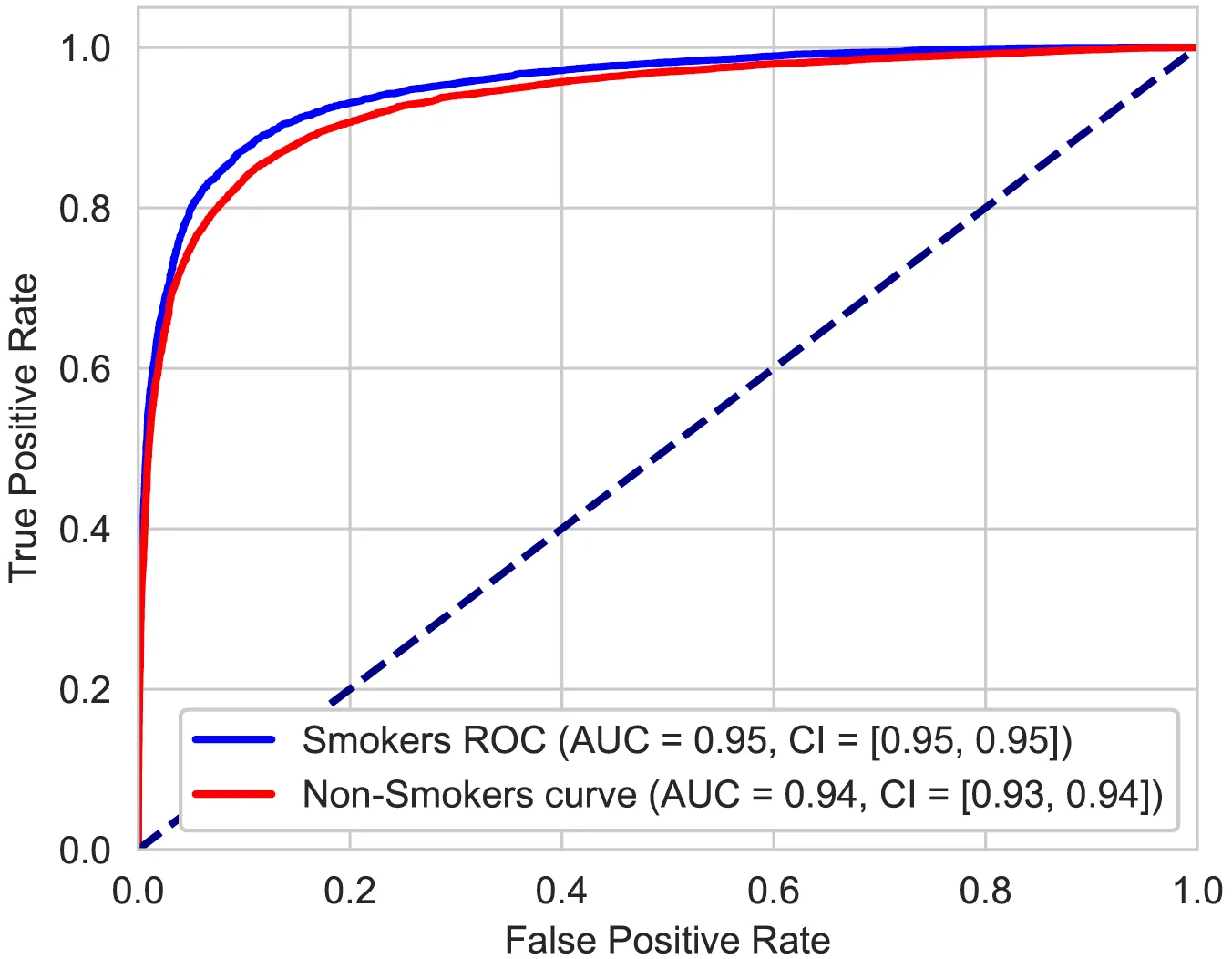
ROC curves for smokers vs. non-smokers. Receiver-operating-characteristic curves for self-reported smokers and non-smokers. Each curve plots the genuine-acceptance rate against the impostor-acceptance rate. Curves farther from the diagonal indicate better genuine/impostor discrimination. Data are based on 8,760 genuine / 8,083 impostor trials for smokers and 12,100 genuine / 10,797 impostor trials for non-smokers. The dashed diagonal indicates chance performance.

**Fig 17:**
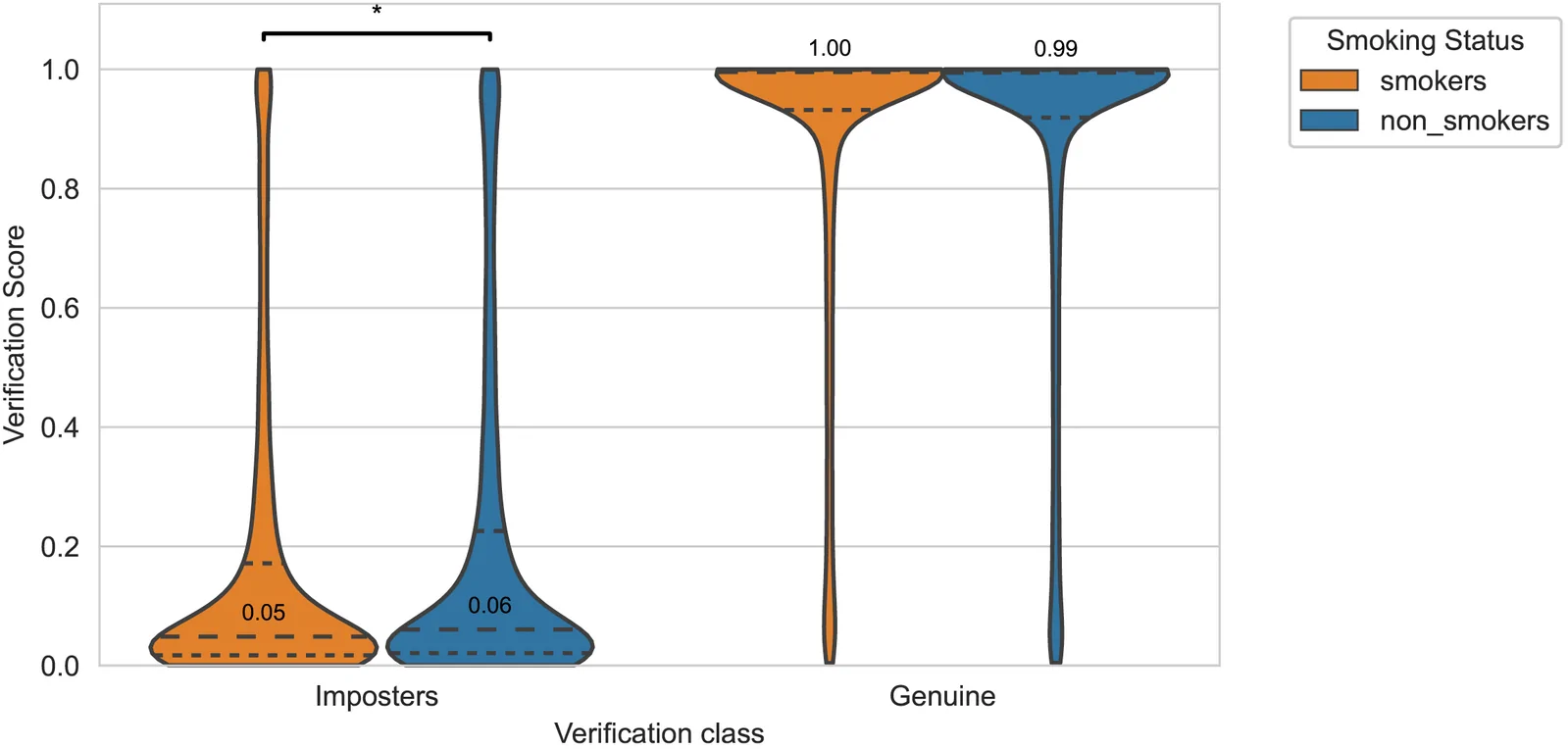
Violin plots showing the distribution of prediction confidence scores by True Target (True or False) and Smoking Status (Smoker vs. Non-Smoker). Violin plots showing similarity-score distributions for genuine (right half of each violin) and impostor (left half) trials in smokers and non-smokers. A greater separation between genuine and impostor score distributions indicates better verification discrimination. An asterisk marks the only significant difference that survived Bonferroni correction (impostor scores, *p* < 0.05); the comparison of genuine scores was non-significant.

Fig 16 shows the performance by Smokers/Non-Smokers, and Fig 17 shows the distribution of verification scores of the groups; significance in brackets corresponds to the statistical test results between verification scores.

A Mann-Whitney U test revealed no significant difference in genuine verification scores between smokers and non-smokers (p = 0.83, z = 0.21), but a significant difference in impostor scores (p = 0.02, z = −2.1), with smokers exhibiting higher scores.

## Discussion

This study evaluated OCAV, an inhalation-triggered verification system, in 99 adult participants. Using participant-specific thresholds, the OCAV achieved 90.1% single trial verification accuracy, which increased to 95.2% using the max score of five aggregated tests. When using a single global threshold, the single-trial accuracy was 88%. The overall AUC was 0.942 and the single trial EER was 12.5%. These findings suggest that under the conditions tested in this study, OCAV generated reliable biometric verification from oral-cavity acoustic reflections. To the best of our knowledge, biometric verification from oral-cavity acoustic reflections has never been reported. We examined variations across sessions, age, and smoking status. The subgroup analyses were exploratory and since some groups were relatively small, so that these results should be interpreted with caution. Across the three weekly visits, genuine-score distributions differed significantly between Visit 1 and Visits 2–3, whereas no significant difference was observed between Visits 2 and 3. The impostor-score distributions did not differ significantly across visits. As shown by the violin plots in Fig 13, the median genuine score remained high across all visits (1.00, 0.99, and 0.99 for Visits 1–3, respectively), compared with a median impostor score of 0.06 at each visit, substantial separation between the genuine and impostor distributions was retained. The ROC curves in Fig 12 nevertheless indicated a measurable session effect, with AUC values of 0.97, 0.94, and 0.92 for Visits 1–3, respectively. These findings support three- week repeatability in the conditions tested here: despite the session-related shift occurring primarily between the first and subsequent visits, the genuine scores remained substantially higher than the impostor scores across all three visits. Longer-term longitudinal studies are needed to further characterize permanence.

In terms of age, there was a small but significant difference in genuine scores (driven by Group 1 vs. Group 3), whereas the impostor comparisons were not statistically significant after Bonferroni correction, indicating little evidence for strong age-related effects. In terms of smoking status, the genuine scores were similar between smokers and non-smokers, but the impostor scores were slightly higher among smokers, the only comparison that remained significant after correction.

User-specific or adaptive thresholding is an established approach in biometric verification and could be incorporated into future OCAV implementations to account for inter-user score variability [29]. The two thresholding strategies implemented here reflect different implementation assumptions. A global threshold is simpler to deploy and scale because the same decision boundary is applied to all users, but it may not account for subject-specific differences in oral acoustic variability. Participant-specific thresholds can partially account for individual score distributions and may improve accuracy, but they require sufficient enrollment data and may reduce generalizability if optimized too closely to the enrollment set. Participant- Participant-specific thresholding affects the decision boundary applied after enrollment and does not in and of itself limit OCAV universality. In this study, the participant-specific thresholds improved single-trial accuracy by about 2% compared to the global threshold. Importantly, these participant-specific results should be interpreted as an upper-bound feasibility estimate under the present protocol, whereas the global-threshold result may better reflect a simpler scalable implementation.

The OCAV system shares conceptual similarities with other excitation-response biometric modalities. For example, in speaker verification, airflow-driven vibration at the larynx provides the excitation, whereas the vocal tract, especially the oral and related cavities, acts like an acoustic filter that shapes the sound spectrum. Its resonances (*formants*) depend on the tract’s anatomy and geometry. These anatomical differences are one of the key reasons why voice has person-specific information and is widely leveraged in speaker verification research [30]. Ear-canal acoustic biometrics inject controlled acoustic signals into the ear canal and characterize the reflected response, leveraging the anatomical stability of the auditory canal [31]. This oral-cavity acoustic approach has several notable advantages over other biometric approaches. Anatomy-driven acoustics have been explored in the ear canal and showed person-specific impulse responses [10,11]. Speaker verification relies on speech and is sensitive to noise and channel mismatch [30,32]. Camera-based biometrics such as face and iris recognition require a clear unobstructed view and consistent lighting [33]. Fingerprint systems typically need finger contact with a clean sensor and clear ridge detail for best results [34]. The OCAV differs from most of these methods by using active sensing, where a brief acoustic audio signal is emitted when a pressure sensor detects inhalation, and the returning acoustic reflections are recorded by a microphone. As a result, the OCAV does not depend on speech input, camera input, or fingerprint capture quality. Under controlled conditions, our test-set AUC (0.942) indicated genuine/impostor separability consistent with our working assumption that anatomic acoustics can encode identity. Direct, head-to-head benchmarking with face/fingerprint/voice was beyond the scope of this study primarily because devices and protocols differ. For this reason, we interpret these findings as feasibility evidence for a complementary, low-effort verification modality. Biometric traits are typically evaluated according to criteria such as universality, distinctiveness, permanence, and resistance to circumvention [1,2]. In the current controlled study, we demonstrated distinctiveness (genuine-impostor separability) and short-term reliability (multi-session stability). Broader validation of large-scale universality, long-term permanence, liveness, spoof resistance, and any future standardization of this biometric trait will require further independent studies.

This study focused on verification accuracy under controlled use. The prototype used here represents laboratory implementation. In practical use, the hygiene requirements would depend on the target device and use model. For personal-use devices such as inhalers or e-cigarettes, the sensing unit would be integrated into a single-user device or used with a personal mouthpiece. For shared-use devices such as breathalyzers, disposable or replaceable mouthpieces, and cleaning protocols would be required. These implementation measures were not evaluated in the current study and require future validation. Because sensing is proximal and inhalation-triggered, the OCAV may be less sensitive to simple audio replay than speech-based methods. However, adversarial testing (e.g., timed replay, mechanical fixtures, or tamper attempts) could not be carried out and will be addressed in future work.

The OCAV technology has significant potential for integration into existing systems to enhance security and public safety. For instance, underage vaping remains common among adolescents (e.g., around 6% of all U.S. middle and high school students reported e‑cigarette use in 2024 [35]). Embedded within e‑cigarettes, the system could deter under-age use. When combined with breath-alcohol testing, mouth-specific verification could reduce cheating, such as having another person blow into the device. This aligns with emerging regulations in the U.S. and E.U. ADAS regulations that require in-vehicle alcohol-interlock systems. An oral-cavity-scanning device could thus enhance road safety by reducing alcohol-related accidents [36,37].

### Strengths and limitations

The OCAV involves a brief interaction that generates an inhalation-triggered acoustic signal. Multi-session sampling per participant enabled assessment of within-person stability across visits. Subgroup analyses were conducted a-priori (age and smoking status) and the data were nearly balanced across groups, supporting fair comparisons. Genuine-impostor evaluations used randomized imposters drawn from the cohort, minimizing selection bias and improving the interpretability of the verification outcomes. From a feature-representation perspective, dimensionality reduction methods such as PCA or autoencoders were not applied in the present study because the feature set was computationally manageable and retained direct correspondence with interpretable acoustic-response and signal-quality descriptors. Future optimization should evaluate whether reduced feature representations can preserve performance while improving efficiency. The limitations include a single site, controlled conditions, and the fact that there was only one hardware prototype. Of the 108 enrolled, the data from 99 participants were analyzed after prespecified exclusions. We did not test robustness to background noise, posture, device variation, or long-term drift, and we did not perform replay/spoofing tests. The other limitations relate to the oral and inhalation-dependent nature of the measurement. Variability in inhalation force, flow, mouthpiece seal, tongue position, saliva, or moisture accumulation may have affected the acoustic response and could increase the false rejection rates outside the laboratory setting. Moisture condensation or saliva accumulation within the mouthpiece or acoustic channel could alter resonance characteristics, dampen acoustic reflections, and affect longitudinal reliability outside the laboratory. Hygiene requirements also depend on the intended use model, personal-use devices may use dedicated mouthpieces, whereas shared devices would require disposable or replaceable mouthpieces and cleaning protocols. Finally, spoofing risks, including artificial oral-cavity models or mechanically timed replay attempts, were not evaluated in this study. These issues require dedicated usability, hardware, hygiene, and adversarial testing before real-world deployment can be considered.

In this study, enrollment and verification were performed under controlled laboratory conditions. Therefore, device-to-device transferability was not evaluated. Use of separate devices at enrollment and verification could introduce differences related to speaker and microphone response, mouthpiece geometry, acoustic coupling, and environmental noise. Practical implementation would require standardized hardware, device calibration, and evaluation under realistic acoustic conditions. The participants were trained to use the device and to inhale in a relatively uniform manner. Therefore, the present results may not fully reflect use by untrained users or by users with more variable inhalation patterns. In real-world settings, differences in inhalation force, flow, mouthpiece seal, or user technique may increase false rejection rates and affect acceptability. These effects should be quantified in future usability and field studies. We also observed visible wear in some of the 3D-printed mouthpieces after repeated attachment-detachment, which could have weakened the seal and may have contributed to small session effects, which were not directly tested. These factors limit generalizability, so that the results should be interpreted as feasibility under the tested conditions rather than a head-to-head benchmark against face, fingerprint, iris, or voice systems.

## Conclusion

The OCAV, a biometric verification device activated during natural inhalation, can reliably verify user identity using low‑cost, commodity hardware. We obtained a single‑trial accuracy of 90.1% (EER ≈ 12.5%) and 95.2% accuracy when aggregating five consecutive samples. Together with the ROC analysis (AUC = 0.942), these results support the feasibility of oral acoustic reflections for user verification under the controlled conditions tested here. The verification device achieved these results in less than 1 sec.

The OCAV system embeds identity verification directly into a user’s natural inhalation process. Each inhalation automatically triggers an acoustic echo recording and on-line verification, requiring no further user actions. This seamless integration illustrates its potential to reduce unauthorized use when incorporated into devices such as e-cigarettes and medical inhalers.

Future work should include testing in noisier and less controlled environments, as well as longitudinal validation over months or years in broader demographic and clinical cohorts. Studies should include direct comparisons with established biometrics, formal spoof-resistance testing, and evaluation of optimized device-integrated prototypes for durability and consistent acoustic coupling. Overall, the findings point to the promise of the OCAV system’s unique combination of speed, convenience, and embedded biometric verification for applications in public health and personalized security.

## Supporting information

S1 Appendix Overview of data flow. Provides a detailed description of the data collection, administrative exclusions, verification-trial pairing, signal-quality filtering, anomaly rejection, and final cohort composition corresponding to Fig 9.
(PDF)
