# Supplementary material for: Biometric verification using oral acoustic reflections
Source: PLoS One. 2026 Sep 25;21(9):e0359160. doi: 10.1371/journal.pone.0359160 (PMC13614562; doi:10.1371/journal.pone.0359160)
Supplement: Supplementary file 1 [file pone.0359160.s001.pdf]

S1 Appendix. Overview of Data Flow

This appendix provides a detailed description of the data flow from initial signal capture through administrative exclusions, verification-trial pairing, signal-quality filtering, anomaly rejection, and final dataset construction corresponding to Fig 9 in the main manuscript.

1. Data capture (raw signals)

   • Enrollment recordings: 108 participants generated 20 inhalations to create a personal profile (108 participants × 20 = 2,160 recordings).

   • Session recordings: Across three weekly test sessions, each participant was asked to perform 80 inhalations per visit, for a theoretical total of 25,920 recordings. (108 participants × 80 inhalations × 3 visits). A total of 23,732 recordings were successfully captured. The remaining 2188 recordings (≈ 8 %) were discarded as a result of brief device-to-PC communication lapses or occasional incorrect device use before they could be written to disk. Thus 23,732 recordings were included in the administrative screening step.

2. Administrative exclusions

   Five participants were deleted from the dataset (4.6 % of the cohort: four for incomplete sessions and one for illness during the study). This left 103 participants, yielding 2,060 profile recordings (103 × 20) and 23,732 session recordings.

3. The pairing table (verification trials)

   A two-column lookup table was generated with *recording ID* and *profile ID*:

   • Each session recording was paired with its owner's profile (genuine trial).

   • The same recording was paired a second time with one randomly sampled profile (seed = 42) from a predefined set of 10 impostor profiles (impostor trial).

   This produced 47, 464 verification trials (2 × 23,732); the profile recordings served

solely as reference templates.

4. Signal-quality and anomaly filtering

   • Variance gate: Recordings whose signal variance lay outside the 1 %–99 % percentile range of the variance distribution (i.e., waveforms that were abnormally quiet or saturated) were discarded.

   • Feature-vector outlier rejection: An unsupervised Isolation-Forest model (200 estimators, contamination ≈ 4 %) detected and removed anomalous samples [1]. Four participants (3.9 % of the remaining cohort) did not have ≥ 12 clean profile recordings and were excluded together with their data.

5. Final verification dataset

   The cleaned pairing table contained 20, 860 genuine trials (52 %) and 18, 880 impostor trials (48 %) totaling 39 740.

Reference

[1] Tony Liu F, Ming Ting K, Zhou Z-H. Isolation Forest ICDM08. Icdm. 2008. Available: https://cs.nju.edu.cn/zhouzh/zhouzh.files/publication/icdm08b.pdf
