# Supplementary material for: Biometric verification using oral acoustic reflections
Source: PLoS One. 2026 Sep 25;21(9):e0359160. doi: 10.1371/journal.pone.0359160 (PMC13614562; doi:10.1371/journal.pone.0359160)
Supplement: Supplementary file 2 [file pone.0359160.s002.docx]

Review for “Biometric verification using oral acoustic reflections”

Comments to Authors:

- A biometric characteristic is related to a human physical or behavioral characteristic, while acoustic reflections are the response of the human body to some external factor.
- This research is based on an artificial setting in which sound waves are transmitted in the oral cavity, and the acoustic response is captured and characterized for verification purposes. It is evident that the raw data collection setup has limited acceptability among users and is also susceptible to serious hygiene issues.
- There should be debate among experts before employing this strategy as a biometric trait. According to the authors, this has never been used as a biometric trait in past research works.
- As there is no prior literature available for this biometric trait, the comparison of this trait with the existing physical and behavioral biometric traits, like finger-vein, face shape, gait, etc., is supposed to be part of the literature. So that the experts may decide whether oral acoustic qualifies as a biometric trait or not.
- To facilitate the experts, for the biometric trait claimed in this manuscript, the degree of the qualities a biometric trait has, like acceptability, universality, uniqueness, reliability, liveliness, hard to counterfeit, etc. are to be reported through some lab or social experiment for which data is collected from common people.
- If some international biometric standardization organization approves oral acoustic as a valid biometric trait, this manuscript may be accepted for publication after the standard review process.
- The specification for hardware resources and software employed is to be mentioned in detail. The computing machine capability and the libraries, toolboxes, and functions employed in experimental simulations are to be mentioned along with the parameters/ hyperparameters details for facilitating researchers interested in confirmation of the reported results and performing comparative analysis.
