# Supplementary material for: Biometric verification using oral acoustic reflections
Source: PLoS One. 2026 Sep 25;21(9):e0359160. doi: 10.1371/journal.pone.0359160 (PMC13614562; doi:10.1371/journal.pone.0359160)
Supplement: Supplementary file 3 [file pone.0359160.s004.docx]

Review for “Biometric verification using oral acoustic reflections”

Round - 2

**Comments to Authors:**

The authors present a novel biometric verification system based on oral cavity acoustics (OCAV).

The authors have largely ignored the first-round comment regarding approval of this biometric trait by an international biometric standardization organization, and then they should proceed with the review process.

However, these observations are to be communicated to the authors.

- The abstract highlights novelty but remains largely descriptive. Please sharpen the novelty claim by explicitly contrasting OCAV with ear-canal and voice biometrics in terms of anatomical stability, spoofing resilience, and deployment feasibility.
- The authors have combined the Introduction section with the related literature section. It is advised to separate both sections. A tabular summary will enhance the readability of the related literature section, listing the methodology employed, main performance metrics reported, and the shortcomings.
- The claimed contributions of the research work are to be mentioned at the end of the related literature section in bulleted form.
- However, the authors have mentioned that sterilization has been performed periodically to handle hygiene issues. The main concern is still there, that is, the serious hygiene issue. As the authors have mentioned, the OCAV profile is generated and recorded when an authorized / unauthorized user inhales using the OCAV system-installed inhaler. Therefore, in real-life environments, there are severe hygiene issues.
- As there will be separate devices used during the capturing of the subject’s OCAV signature at the time of enrollment (in the lab) and at the time of verification/identification (noisy real-life environment). There are chances of variations in the captured OCAV signatures at the enrollment and verification stages. This issue is to be discussed, and an explanation is required to handle it.
- The authors have mentioned that during the pre-training session, the participating subjects were trained regarding the proper usage of the inhaler. Hence, the margin of error and rejection rate for genuine users are expected to be higher when implemented in real life. Further, this uniform inhalation requirement results in limited acceptability, universality, and reliability.
- The approval letter (approval no. 467-3) is required to be presented in the Appendix.
- The 342-dimensional feature vector is described but not justified. Please explain why this dimensionality was chosen and whether dimensionality reduction (e.g., PCA, autoencoders) was considered to improve efficiency.
- The function of the Isolation Forest model requires explanation when employed to label the outlier feature vectors.
- The LightGBM classifier is trained on an in-house dataset. Clarify whether cross-validation was used and discuss risks of overfitting given the relatively small participant pool. This will reassure readers about methodological rigor.
- The authors have reported both global and participant-specific thresholds. Please expand the discussion of implications for deployment, particularly the trade-off between generalizability and personalization, and scalability concerns.
- These figures have the following concerns:
  - Fig. 2 (D) schematic diagram: needs clearer labeling of components and signal flow.
  - Fig. 5 transfer functions: there is only one transmitted and reflected signal, while the caption states ‘Transfer functions of two different participants’. No transfer function h(t) is defined in this figure in some mathematical equation form. This figure is to be revised.
  - ROC and violin plots (Figs. 10–17): captions and text should explain what the differences mean, not just present curves.
- The discussion underplays limitations. Please add explicit mention of inhalation variability, spoofing risks (e.g., artificial oral cavities), and hygiene challenges, along with preliminary mitigation strategies.
- The conclusion section must justify the claimed contributions by referring to the presented experimental results in the form of performance metrics or ROC graphs.
- The conclusion could be enriched by suggesting next steps: testing in noisy environments, integration into commercial devices, and longitudinal studies to assess stability over months/years. This would give the paper a stronger translational impact.
