# Supplementary material for: Biometric verification using oral acoustic reflections
Source: PLoS One. 2026 Sep 25;21(9):e0359160. doi: 10.1371/journal.pone.0359160 (PMC13614562; doi:10.1371/journal.pone.0359160)
Supplement: Supplementary file 4 [file pone.0359160.s005.docx]

**Response to Editor and Reviewers**

Manuscript Title: *Biometric verification using oral acoustic reflections*
Journal: PLOS ONE

Dear Editor,

We thank you for your careful evaluation of our manuscript and for your detailed guidance. We have carefully revised the manuscript and submission materials in accordance with all editorial and reviewer comments. Below we provide point-by-point responses.

**Editor Comments**

**Editor Comment 1:**

Please ensure that your manuscript meets PLOS ONE’s style requirements.

**Response:**
We have carefully reviewed the manuscript to ensure full compliance with PLOS ONE formatting and file-naming requirements. All files have been updated accordingly.

**Editor Comment 2:**

Please note that PLOS One has specific guidelines on code sharing for submissions in which author-generated code underpins the findings in the manuscript. In these cases, we expect all author-generated code to be made available without restrictions upon publication of the work.

**Response:**
Upon publication, we will deposit in a stable public repository all materials necessary to reproduce the findings reported in the manuscript. These include:

- The trained LightGBM model used for verification
- The full anonymized 242-dimensional comparison feature matrix
- The complete score matrix for all genuine and impostor comparisons
- The corresponding genuine/impostor labels
- The raw de-identified oral-acoustic recordings
- Scripts required to reproduce all reported performance metrics (accuracy, ROC curves, AUC, and EER)
- Code for loading and processing the released raw audio files

These materials enable independent replication of all statistical analyses and performance results presented in the manuscript.
The proprietary feature-extraction development framework and original model training procedure are not included; however, retraining is not required to reproduce the published results, as the trained model and all evaluation inputs are provided.
Commercial affiliations do not restrict access to any materials necessary to reproduce the study’s conclusions.

**Editor Comment 3.1:**

We note that one or more of the authors are employed by a commercial company:Echo-ID. Please provide an amended Funding Statement declaring this commercial affiliation, as well as a statement regarding the Role of Funders in your study.

**Response:**

**Funding Statement**

This study was supported by Echo-ID Ltd., which provided institutional funding to Ben-Gurion University of the Negev for the conduct of this research. Echo-ID Ltd. also provided support in the form of salaries for authors A.K., D.T., E.H., and R.R., and provided the OCAV device used in this study.
The study was designed collaboratively by academic investigators and co-author collaborators. The funder did not have any additional role in the independent data analysis, decision to publish, or preparation of the manuscript beyond the involvement of the named authors as co-investigators. The specific roles of these authors are articulated in the ‘Author Contributions’ section.

**Editor Comment 3.2:**

Please also provide an updated Competing Interests Statement declaring this commercial affiliation along with any other relevant declarations relating to employment, consultancy, patents, products in development, or marketed products, etc.

**Response:**

**Competing Interests Statement**

The authors declare the following competing interests: Echo-ID Ltd. provided institutional funding for this study. Amit Kam, Daniel Tubiana, Eran Hochstadter, and Roi Ram are employees of Echo-ID Ltd. The OCAV device evaluated in this study was developed by Echo-ID Ltd. Echo-ID Ltd. holds patent protection related to the OCAV system. None of the authors are named inventors on the patent.

This does not alter our adherence to PLOS ONE policies on sharing data and materials.

**Editor Comment 4:**

a) If there are ethical or legal restrictions on sharing a de-identified data set, please explain them in detail (e.g., data contain potentially identifying or sensitive patient information, data are owned by a third-party organization, etc.) and who has imposed them (e.g., a Research Ethics Committee or Institutional Review Board, etc.). Please also provide contact information for a data access committee, ethics committee, or other institutional body to which data requests may be sent.

b) If there are no restrictions, please upload the minimal anonymized data set necessary to replicate your study findings to a stable, public repository and provide us with the relevant URLs, DOIs, or accession numbers. For a list of recommended repositories

**Response:**

We thank the Editor for this clarification.
All data necessary to replicate the findings of this study will be publicly available in a stable repository (DOI to be provided prior to publication). This includes the anonymized 242-dimensional comparison feature matrix, the trained LightGBM model, genuine/impostor labels, and scripts required to reproduce all reported performance metrics. These materials are sufficient to independently replicate all statistical analyses and conclusions presented in the manuscript.
The raw de-identified oral acoustic recordings are not required to reproduce the published findings. However, because these recordings contain acoustic signatures derived from individual anatomical structures that may have the potential to function as biometric identifiers, access to the raw audio files will be provided upon reasonable request to qualified researchers. Requests may be directed to: [data-access@echo-id.com](mailto:data-access@echo-id.com). Access will be granted for legitimate research purposes in a manner consistent with institutional and ethical guidelines.

**Editor Comment 5:**

Provide amended data availability statement.

**Response:**

**Data Availability Statement**

All data necessary to replicate the findings of this study will be publicly available in a stable repository (DOI to be provided upon publication). This includes the anonymized 242-dimensional comparison feature matrix, the trained LightGBM model, genuine/impostor labels, and scripts required to reproduce all reported performance metrics.

The raw de-identified oral acoustic recordings are not required to reproduce the published results but will be made available to qualified researchers upon reasonable request. Requests should be directed to data-access@echo-id.com. Access will be provided in accordance with institutional and ethical guidelines.

**Editor Comment 6:**

We notice that your supplementary figures are uploaded with the file type 'Figure'. Please amend the file type to 'Supporting Information'. Please ensure that each Supporting Information file has a legend listed in the manuscript after the references list.

**Response:**

The supplementary file was a document file (.docx) and not a figure we have change this to a pdf file. We have amended the file type in the submission system from “Figure” to “Supporting Information.”
We have also ensured that the corresponding legend for the Supporting Information file is included in the manuscript immediately following the References section, in accordance with PLOS ONE guidelines.

**Responses to Reviewers**

We thank the reviewers for their careful evaluation of our manuscript and for their constructive comments. We have revised the manuscript accordingly and provide detailed responses below.

**Reviewer #1 – Comment 1**

A biometric characteristic is related to a human physical or behavioral characteristic, while acoustic reflections are the response of the human body to some external factor.

**Response:**

We note that several established biometric modalities, like speaker verification and ear-canal acoustic biometrics, rely on excitation response measurements of stable anatomical structures. We have clarified this point in the discussion third paragraph.

**Reviewer #1 – Comment 2**

This research is based on an artificial setting in which sound waves are transmitted in the oral cavity, and the acoustic response is captured and characterized for verification purposes. It is evident that the raw data collection setup has limited acceptability among users and is also susceptible to serious hygiene issues.

**Response:**

The present study, recruited above 100 real participants, employed a laboratory prototype to evaluate verification performance under controlled conditions. In practical applications, the OCAV sensing unit is intended to be embedded within personal-use oral devices (e.g., inhalers, e-cigarettes, breathalyzers), where hygiene management and individual mouth contact are already standard. We have clarified this point in the Discussion.

**Reviewer #1 – Comments 3-5**

There should be debate among experts before employing this strategy as a biometric trait. According to the authors, this has never been used as a biometric trait in past research works.

As there is no prior literature available for this biometric trait, the comparison of this trait with the existing physical and behavioral biometric traits, like finger-vein, face shape, gait, etc., is supposed to be part of the literature. So that the experts may decide whether oral acoustic qualifies as a biometric trait or not.

To facilitate the experts, for the biometric trait claimed in this manuscript, the degree of the qualities a biometric trait has, like acceptability, universality, uniqueness, reliability, liveliness, hard to counterfeit, etc. are to be reported through some lab or social experiment for which data is collected from common people.

**Response:**

We agree that new biometric modalities should be interpreted within established biometric evaluation frameworks. In the revised Discussion, we now explicitly position OCAV relative to classical biometric criteria (e.g., universality, distinctiveness, permanence, and resistance to circumvention [1,2]) and clarify the scope of the present work.

This study was designed as a controlled feasibility evaluation. We demonstrate distinctiveness (genuine–impostor separability) and short-term reliability (multi-session stability) under controlled conditions. Broader validation of large-scale universality, long-term permanence, formal liveness testing, spoof resistance, and user acceptability would require additional independent investigation beyond the scope of the current study.

**Reviewer #1 – Comment 6**

If some international biometric standardization organization approves oral acoustic as a valid biometric trait, this manuscript may be accepted for publication after the standard review process.

**Response:**

We respectfully note that formal standardization typically follows scientific validation and broader independent evaluation, rather than preceding it. The present manuscript reports a controlled feasibility study of a novel verification approach. Publication of such early-stage research enables further independent validation.

**Reviewer #1 – Comment 7**

The specification for hardware resources and software employed is to be mentioned in detail. The computing machine capability and the libraries, toolboxes, and functions employed in experimental simulations are to be mentioned along with the parameters/ hyperparameters details for facilitating researchers interested in confirmation of the reported results and performing comparative analysis.

**Response:**

We have expanded the Methods section to clarify the computational environment and implementation details. The manuscript now specifies the computing platform, software environment, Python version and libraries (Performance metrics & statistical analyses subchapter), and the key model hyperparameters used, LightGBM and Isolation Forest (Verification process subchapter).

**Reviewer #2 – Comment 1**

Classical biometric traits are inherently linked to stable human physiological or behavioral characteristics. In contrast, acoustic reflections primarily represent the body’s response to externally injected stimuli, which raises questions about their classification as a true biometric attribute.

**Response:**
As addressed in response to Reviewer #1 (Comment 1), we have clarified in the revised Discussion that several established biometric modalities (e.g., speaker verification and ear-canal acoustics) rely on excitation–response measurements of stable anatomical structures. We emphasize that the OCAV system measures acoustic reflections determined by the subject’s stable oral-cavity geometry.

**Reviewer #2 – Comment 2**

The proposed methodology relies on an artificial acquisition setup where sound waves are actively introduced into the oral cavity and the resulting acoustic response is analyzed for identity verification. Such a data acquisition process may face significant challenges in terms of user comfort, practical acceptability, and hygiene, particularly in real-world deployment scenarios.

**Response:**
 As clarified in response to Reviewer #1 (Comment 2), the present study employed a laboratory prototype to evaluate verification accuracy under controlled conditions. In the revised Discussion, we now explicitly state that the OCAV sensing unit is intended for integration into personal-use oral devices (e.g., inhalers, e-cigarettes, breathalyzers), where individual mouth contact and hygiene management are already standard.

**Reviewer #2 – Comment 3**

Given that the authors claim novelty in introducing oral acoustic response as a biometric modality, a broader discussion within the biometric research community appears necessary before considering its adoption. To the best of the reviewer’s knowledge, this modality has not been formally established or validated as a biometric trait in prior studies.

**Response:**
The present manuscript reports a controlled feasibility evaluation of a novel verification modality. In the revised Discussion, we explicitly position OCAV within established biometric evaluation criteria and clarify the scope of the current study. We emphasize that broader validation, replication, and community-level adoption would require further independent investigation beyond the scope of this initial feasibility work.

**Reviewer #2 – Comment 4**

The manuscript should provide comprehensive details of the experimental setup, including hardware specifications and software resources. Information regarding the computing platform, employed libraries or toolboxes, and all relevant parameters and hyperparameters used in simulations should be explicitly stated to ensure reproducibility and facilitate comparative studies by future researchers.

**Response:**
The Methods section has been expanded to clarify the computational environment and implementation details. The revised manuscript now specifies the computing platform, Python version, software libraries used, and the key model hyperparameters (e.g., Isolation Forest and LightGBM settings). No proprietary toolboxes were employed. We believe these additions improve transparency and facilitate reproducibility and comparative evaluation.

**Reviewer #2 – Comment 5**
To support the proposed claim, an evaluation of standard biometric properties such as universality, acceptability, uniqueness, permanence, reliability, liveness detection, and resistance to spoofing should be conducted. Ideally, these properties should be quantified through controlled laboratory studies or socially representative experiments involving data collected from a diverse population.

**Response:**
The present work was designed as a controlled feasibility study rather than a comprehensive biometric trait validation program.

In this study, we quantitatively evaluated distinctiveness (genuine–impostor separability), short-term reliability (multi-session stability), and subgroup consistency (age and smoking status) within the studied cohort.

Comprehensive evaluation of large-scale universality across diverse populations, long-term permanence, formal liveness detection, spoof resistance, and real-world acceptability would require broader, multi-center validation studies and dedicated adversarial testing. These investigations are important future directions but are beyond the scope of the present study.

**Reviewer #2 – Comment 6**
The authors should consider referencing prior studies on respiratory and airway acoustic/impedance modeling, where anatomical and biomechanical properties of the oral–respiratory system were shown to produce distinctive and repeatable acoustic responses. In particular, respiratory-signal-based modeling approaches used for inhalation impedance estimation and respiratory pattern analysis could provide additional context supporting the uniqueness and stability of oral acoustic reflections used for biometric verification. I suggest authors cite this: 'Spirometer and sEMG respiratory patterns for clinical decision support system'. ( doi: 10.1109/I2MTC53148.2023.10175925 )

**Response:**
We thank the reviewer for the suggestion. The manuscript already cites prior studies that directly address acoustic modeling of the vocal tract. The suggested paper focuses mainly on respiratory signal analysis rather than oral acoustic reflections. To keep the references closely aligned with the scope of this study, we retained citations that are directly relevant to the acoustic approach used here.

**Reviewer #2 – Comment 7**
Since there is no established literature on oral acoustics as a biometric modality, the manuscript would benefit from a comparative discussion with well-known physical and behavioral biometrics (e.g., fingerprint, finger-vein, facial structure, gait). Such a comparison could help clarify whether oral acoustic characteristics meet the fundamental criteria required of a biometric trait.

**Response:**
In the revised Discussion, we have added a concise comparison positioning OCAV within established biometric evaluation criteria (e.g., universality, distinctiveness, permanence, resistance to circumvention) and clarified the scope of the present feasibility study. We believe this addition provides appropriate context relative to well-known biometric modalities without extending beyond the study’s objectives.

**Reviewer #2 – Comment 8**

Acceptance of oral acoustic response as a valid biometric modality would be significantly strengthened if it were endorsed or standardized by a recognized international biometric standardization body. Without such validation, the contribution remains preliminary.

**Response:**
As noted in our response to Reviewer #1 regarding standardization, the present work is intended as a controlled feasibility study. Broader validation and potential endorsement by standardization bodies would require further independent investigation beyond the scope of the current manuscript.

**Closing Statement**

We sincerely thank the Editor and Reviewers for their thoughtful and constructive feedback, which has helped improve the clarity, transparency, and positioning of this manuscript. We believe the revised version addresses all concerns raised and meets PLOS ONE’s policies and reporting standards.

We appreciate your time and consideration.

Sincerely,
Amit Kam
(on behalf of all authors)
