# Supplementary material for: Biometric verification using oral acoustic reflections
Source: PLoS One. 2026 Sep 25;21(9):e0359160. doi: 10.1371/journal.pone.0359160 (PMC13614562; doi:10.1371/journal.pone.0359160)
Supplement: Supplementary file 5 [file pone.0359160.s006.docx]

Review for “Biometric verification using oral acoustic reflections”

Round - 3

Comments to Authors:

In response to the previous round comments, the authors have made significant changes. These observations are made from the revised manuscript:

- The Authors stated that the study was a controlled feasibility study rather than a standardized or fully validated biometric modality.
- Enhancements have been made to the abstract and introduction, which include contrasts between OCAV and existing biometric approaches, and a comparative literature table.
- Bulleted contributions, ethics approval paperwork, and better labeling of figures have been added.
- Increased discussion of limitations (hygiene issues, inhalation variation, spoofing risk, device variation) has been added.
- Technical details have been explained, including the 342-dimensional feature vector, Isolation Forest role, LightGBM training/testing separating and thresholding strategies.
- The claimed contributions have been linked to the derived conclusions and set future directions.

These points of improvement are to be communicated to the authors.

1. The authors have included this sentence in the main contributions (Identify current limitations related to controlled acquisition, inhalation variability, device consistency, hygiene, and the need for future validation). This sentence should not be written in the main contributions but should be mentioned in the conclusion and discussion sections only.
2. The discussion from line number 398 to 414 of the revised manuscript gives the impression that the genuine scores vary between trial sessions. Hence, OCAV has low permanence. Permanence is a characteristic required for a human quality to be qualified as a biometric trait. Some justification for this should be included in the manuscript.
3. The authors should clarify that using participant-specific thresholds won’t affect the universality of the OCAV inversely.
4. The given reason for the dismissal of PCA/autoencoders as “not applicable because manageable” is convincing. If there is a better reason, for example, interpretability or reproducibility, then there will be more confidence in the feature set chosen.
5. The manuscript should be checked for English language mistakes employing some human language expert or specialized language software in consent with the authors.
