# Supplementary material for: Biometric verification using oral acoustic reflections
Source: PLoS One. 2026 Sep 25;21(9):e0359160. doi: 10.1371/journal.pone.0359160 (PMC13614562; doi:10.1371/journal.pone.0359160)
Supplement: Supplementary file 6 [file pone.0359160.s007.docx]

**Response to Editor and Reviewers**

**Manuscript Title: *Biometric verification using oral acoustic reflections***

**Journal: PLOS ONE**

Dear Dr. Forero,

We thank you and the reviewers for the careful evaluation of our manuscript and for the opportunity to submit a revised version. We have revised the manuscript to address all the issues raised in the decision letter and in the reports of Reviewer #1 and Reviewer #3, with particular attention to methodological clarity, feasibility-study framing, reproducibility, and limitations related to real-world deployment.

In accordance with the Editorial Office instruction, we have disregarded the report of Reviewer #2 and do not provide responses to that reviewer. The point-by-point responses below therefore address the comments requiring response from the Academic Editor, Reviewer #1, and Reviewer #3 only.

In the revised manuscript, we have clarified the positioning of OCAV as a controlled feasibility study rather than a deployment-ready or fully validated biometric modality. We have also expanded the discussion of train/test separation, thresholding, feature-vector terminology, outlier filtering, inhalation variability, hygiene considerations, hardware and environmental limitations, moisture/saliva effects, and the need for future validation before any real-world implementation.

**Responses to Reviewers**

We thank the reviewers for their careful evaluation of our manuscript and for their constructive comments. We have revised the manuscript accordingly and provided detailed responses below.

**Reviewer #1 – Opening comment**

The authors have largely ignored the first-round comment regarding approval of this biometric trait by an international biometric standardization organization, and then they should proceed with the review process.

**Response:**

We thank the reviewer for this comment. Oral acoustic reflection is presented here as a new verification approach, and to the best of our knowledge it has not previously been evaluated as a biometric trait. The manuscript is therefore framed as a controlled feasibility study, not as a standardized or fully validated biometric modality. To clarify this scope, we added a sentence to the Discussion stating that any future standardization of this biometric trait would require further independent validation.

**Reviewer #1 – Comment 1**

The abstract highlights novelty but remains largely descriptive. Please sharpen the novelty claim by explicitly contrasting OCAV with ear-canal and voice biometrics in terms of anatomical stability, spoofing resilience, and deployment feasibility.

**Response:**

We revised the Abstract to more clearly position OCAV relative to voice and ear-canal acoustic biometrics. The revised text clarifies that OCAV uses an active acoustic response from the oral cavity and is designed for oral substance-exchange devices and can be integrated in systems that already involve oral cavity contact.

**Reviewer #1 – Comment 2**

The authors have combined the Introduction section with the related literature section. It is advised to separate both sections. A tabular summary will enhance the readability of the related literature section, listing the methodology employed, main performance metrics reported, and the shortcomings.

**Response:**

We revised the Introduction to more clearly separate the background literature from the study objective. Specifically, we divided the section into clearer subsections, including a “Related literature” and “Study objective” subsections. We added a summary table comparing relevant biometric approaches by methodology, reported performance context, and main limitations relevant to OCAV. This improves readability while preserving the focused feasibility-study structure of the manuscript.

**Reviewer #1 – Comment 3**

The claimed contributions of the research work are to be mentioned at the end of the related literature section in bulleted form.

**Response:**

We added a bulleted summary of the study contributions at the end of the Introduction section. The new text summarizes the main contributions of the study, including the proposed oral acoustic verification approach, the experimental workflow, the multi-session performance evaluation, and the limitations requiring future validation.

**Reviewer #1 – Comment 4**

However, the authors have mentioned that sterilization has been performed periodically to handle hygiene issues. The main concern is still there that is, the serious hygiene issue. As the authors have mentioned, the OCAV profile is generated and recorded when an authorized / unauthorized user inhales using the OCAV system-installed inhaler. Therefore, in real-life environments, there are severe hygiene issues.

**Response:**

We revised the Discussion, strength and limitations to acknowledge hygiene as a real-life implementation issue. The revised text now clarifies that hygiene requirements depend on the intended use model, with dedicated mouthpieces for personal-use devices and disposable or replaceable mouthpieces with cleaning protocols for shared-use devices.

**Reviewer #1 – Comment 5**

As there will be separate devices used during the capturing of the subject’s OCAV signature at the time of enrollment (in the lab) and at the time of verification/identification (noisy real-life environment). There are chances of variations in the captured OCAV signatures at the enrollment and verification stages. This issue is to be discussed, and an explanation is required to handle it.

**Response:**

We revised the limitations section to address possible variation between enrollment and verification devices. The revised text clarifies that device-to-device transferability was not evaluated in the present controlled study, and that separate enrollment and verification devices could introduce variation related to speaker and microphone response, mouthpiece geometry, acoustic coupling, and environmental noise. We also added that practical implementation would require standardized hardware, device calibration, and evaluation under realistic acoustic conditions.

**Reviewer #1 – Comment 6**

The authors have mentioned that during the pre-training session, the participating subjects were trained regarding the proper usage of the inhaler. Hence, the margin of error and rejection rate for genuine users are expected to be higher when implemented in real life. Further, this uniform inhalation requirement results in limited acceptability, universality, and reliability.

**Response:**

We expanded the limitations section to address the effect of participant training and uniform inhalation. The revised text clarifies that participants were trained to use the device and inhale in a relatively uniform manner, and that the present results may not fully reflect use by untrained users or users with more variable inhalation patterns. We also added that differences in inhalation force, flow, mouthpiece seal, or user technique may affect false rejection rates and should be evaluated in future usability and field studies.

**Reviewer #1 – Comment 7**

The approval letter (approval no. 467-3) is required to be presented in the Appendix

**Response:**

The ethics approval letter for approval no. 467-3 has been added as Supporting Information.

**Reviewer #1 – Comment 8**

The 342-dimensional feature vector is described but not justified. Please explain why this dimensionality was chosen and whether dimensionality reduction (e.g., PCA, autoencoders) was considered to improve efficiency.

**Response:**

We revised the Feature extraction section to justify the 342-dimensional feature vector. The revised text explains that the vector was generated by a fixed feature-extraction procedure summarizing spectral-response patterns, early reflection information, frequency-banded residual-noise characteristics, and global signal-quality measures. We also clarified under the strength and limitation section, that PCA or autoencoder-based dimensionality reduction was not applied because the feature set was computationally manageable and retained interpretable acoustic-response descriptors.

**Reviewer #1 – Comment 9**

The function of the Isolation Forest model requires explanation when employed to label the outlier feature vectors.

**Response:**

We revised the Enrollment process section to explain the function of the Isolation Forest model. The added text clarifies that it was used as an unsupervised quality-control step applied to recording-quality features to identify atypical recordings before profile formation.

**Reviewer #1 – Comment 10**

The LightGBM classifier is trained on an in-house dataset. Clarify whether cross-validation was used and discuss risks of overfitting given the relatively small participant pool. This will reassure readers about methodological rigor.

**Response:**

We revised the Classification Model section to clarify the training and evaluation separation. The added text states that the pre-existing LightGBM training dataset and the 108-participant evaluation cohort were subject-disjoint, and that no cross-validation folds from the present cohort were used for classifier training.

**Reviewer #1 – Comment 11**

The authors have reported both global and participant-specific thresholds. Please expand the discussion of implications for deployment, particularly the trade-off between generalizability and personalization, and scalability concerns.

**Response:**

We expanded the Discussion section to clarify the implications of the global and participant-specific threshold analyses. The revised text explains that a global threshold is simpler to deploy and scale because the same decision boundary is applied to all users, but it may not account for subject-specific differences in oral acoustic variability. We also clarify that participant-specific thresholds can partially account for individual score distributions and may improve accuracy but require sufficient enrollment data and may reduce generalizability if optimized too closely to the enrollment set. We therefore state that the participant-specific results should be interpreted as an upper-bound feasibility estimate under the present protocol, whereas the global-threshold result may better reflect a simpler scalable implementation.

**Reviewer #1 – Comment 12**

Fig. 2 (D) schematic diagram: needs clearer labeling of components and signal flow.

Fig. 5 transfer functions: there is only one transmitted and reflected signal, while the caption states ‘Transfer functions of two different participants’. No transfer function h(t) is defined in this figure in some mathematical equation form. This figure is to be revised.

ROC and violin plots (Figs. 10–17): captions and text should explain what the differences mean, not just present curves.

**Response:**

We revised the figures and captions to improve clarity. For Fig. 2(D), we added labels and arrows identifying the main components including the speaker, microphone, waveguide/acoustic path, pressure sensor, seal and battery.

For Fig. 5, the reviewer’s concern resulted from an error in the figure numbering. We corrected the figure numbering and verified that the correct figure is now included. The corrected Fig. 5 shows the estimated transfer-function magnitude responses from two representative participants, consistent with the transfer-function definition provided in the Methods section.

We also revised the captions for the ROC and violin plots (Figs. 10–17) to better explain the interpretation of the plotted results, including, score-distribution separation, and corrected statistical significance markers.

**Reviewer #1 – Comment 13**

The discussion underplays limitations. Please add explicit mention of inhalation variability, spoofing risks (e.g., artificial oral cavities), and hygiene challenges, along with preliminary mitigation strategies.

**Response:**

We expanded the limitations section to address these issues explicitly. The revised text now discusses inhalation variability, mouthpiece seal, tongue position, saliva or moisture effects, hygiene requirements for personal-use and shared devices, and spoofing risks such as artificial oral-cavity models or mechanically timed replay attempts. We also clarified that these issues were not evaluated in the present controlled study and require dedicated future testing before real-world deployment can be considered.

**Reviewer #1 – Comment 14**

The conclusion section must justify the claimed contributions by referring to the presented experimental results in the form of performance metrics or ROC graphs.

**Response:**

We revised the Conclusion to link the study contribution more directly to the experimental results. The revised text now refers to the single-trial accuracy, five-sample aggregation accuracy, EER, and ROC/AUC results, and frames these findings as feasibility evidence under the controlled conditions tested in this study.

**Reviewer #1 – Comment 15**

The conclusion could be enriched by suggesting next steps: testing in noisy environments, integration into commercial devices, and longitudinal studies to assess stability over months/years. This would give the paper a stronger translational impact.

**Response:**

We revised the Conclusion to expand the future-work directions. The revised text now identifies testing in noisier and less controlled environments, longitudinal validation over months or years, anti-spoofing evaluations, and evaluation of optimized device-integrated prototypes as necessary next steps.

**Reviewer #3 – Comment 1**

In the "Classification Model" section, the authors state: "We trained the LightGBM classifier on a pre-existing in-house dataset of 20,000 comparison vectors...". It is imperative to explicitly clarify the demographic origin of this "in-house dataset." Specifically, the authors must state whether the subjects in this training dataset are entirely mutually exclusive from the 108 subjects used in the testing cohort. If there is any subject overlap between the pre-existing training set and the test set, this would introduce a substantial risk of data leakage and could materially inflate the reported AUC and accuracy estimates. Please add a sentence or paragraph explicitly defining the separation between the training and testing sets to assure the readers of the model's generalizability and statistical rigor.

**Response:**

We revised the Classification Model section to explicitly clarify the separation between the pre-existing LightGBM training dataset and the evaluation cohort used in the present study. The added text states that the participants used to generate the pre-existing training dataset were not included in the 108-participant evaluation cohort reported in this study. Thus, the classifier training set and the present evaluation set were subject-disjoint. We also clarified that model development and parameter selection were performed before evaluation on the present cohort, and that data from the present study cohort were not used for classifier training. These clarifications were added to address the possibility of subject-level data leakage and to make the training/testing separation transparent.

**Reviewer #3 – Comment 2**

Fluid Dynamics and Inhalation Variance
The system relies on a pressure drop during inhalation to trigger the acoustic chirp. Under controlled laboratory conditions, subjects were trained to breathe uniformly. However, in real-world applications (e.g., impaired individuals using breathalyzers, or patients with varying respiratory capacities), inhalation force and velocity will fluctuate significantly. Such variations could affect the measured acoustic transfer function and therefore the stability of verification performance in real-world use.
Did the training or testing protocols evaluate intra-subject variance across different inhalation intensities? If this variable was controlled or assumed constant, it must be explicitly acknowledged as a constraint in the "Strengths and limitations" section, as natural respiratory variance could affect the stability of the transfer function in field deployments.

**Response:**

We agree with the reviewer that inhalation variability is an important factor for field use. The present study did not include a dedicated sub-protocol in which inhalation force or flow rate was systematically varied within participants. Instead, participants were trained to use the device in a relatively uniform manner under controlled laboratory conditions. We therefore revised the Strengths and limitations section to explicitly acknowledge this constraint. The revised text states that variability in inhalation force, flow, mouthpiece seal, tongue position, saliva, or moisture accumulation may affect the measured acoustic response and could increase false rejection rates outside the laboratory setting. We also added that future usability and field studies should quantify these effects in untrained users and in users with more variable inhalation patterns.

**Reviewer #3 – Comment 3**

Environmental Degradation of the Acoustic Channel
In the limitations section, the authors appropriately mention the mechanical wear of the 3D-printed mouthpieces. However, for a device handling oral substance-exchange, acoustic sensors are highly susceptible to micro-environmental changes.

Please add a brief acknowledgment in the limitations section regarding the potential impact of moisture condensation or saliva accumulation within the acoustic channel over time. These factors can alter the resonance and dampen the acoustic reflections, which would be important to consider for longitudinal reliability outside the laboratory.

**Response:**

We revised the Strengths and limitations section to explicitly address possible micro-environmental changes in the acoustic channel. The revised text now states that saliva or moisture accumulation may affect the measured acoustic response, and we further clarify that such effects could alter resonance characteristics, dampen acoustic reflections, and affect longitudinal reliability outside the laboratory. We also note that these effects were not evaluated in the present controlled feasibility study and should be assessed in future studies using optimized, device-integrated prototypes.

**Closing Statement**

We sincerely thank the Editor and Reviewers for their thoughtful and constructive feedback, which has helped improve the clarity, transparency, and positioning of this manuscript. We believe the revised version addresses all concerns raised and meets PLOS ONE’s policies and reporting standards.

We appreciate your time and consideration.

Sincerely,
Amit Kam
(on behalf of all authors)
