# Supplementary material for: Biometric verification using oral acoustic reflections
Source: PLoS One. 2026 Sep 25;21(9):e0359160. doi: 10.1371/journal.pone.0359160 (PMC13614562; doi:10.1371/journal.pone.0359160)
Supplement: Supplementary file 7 [file pone.0359160.s008.docx]

Review for “Biometric verification using oral acoustic reflections”

Round - 4

**Comments to Authors:**

In response to the previous round comments, the authors have made significant changes. All the communicated observations have been addressed in the revised manuscript.

However, I still feel that it is required to perform longer span longitudinal study to evaluate the degree of OCAV permanence and other biometric characteristics. This point has already been acknowledged by the authors at line number 421 of the revised manuscript.

The manuscript has also been reviewed by human English language expert. Therefore, in my opinion, the manuscript may be accepted in the current form.
