# Supplementary material for: Biometric verification using oral acoustic reflections
Source: PLoS One. 2026 Sep 25;21(9):e0359160. doi: 10.1371/journal.pone.0359160 (PMC13614562; doi:10.1371/journal.pone.0359160)
Supplement: Supplementary file 8 [file pone.0359160.s009.docx]

**Response to Editor and Reviewers**

**Dr. Forero**

**PLOS ONE**

August 15,2026

**Revision of: *Biometric verification using oral acoustic reflections***

Dear Dr. Forero,

Thank you for the opportunity to revise our manuscript entitled “Biometric verification using oral acoustic reflections.” We appreciate the positive evaluation of the revised manuscript and the additional minor comments provided by Reviewer #1. We have addressed each of the remaining comments and revised the manuscript accordingly. Our point-by-point responses are provided below, and all manuscript changes are identified in the track-changes version.

**Responses to Reviewers**

We thank the reviewers for their careful evaluation of our manuscript and for their constructive comments. We have revised the manuscript accordingly and provide detailed responses below.

**Reviewer #1 – Comment 1**

The authors have included this sentence in the main contributions: “Identify current limitations related to controlled acquisition, inhalation variability, device consistency, hygiene, and the need for future validation.” This sentence should not be written in the main contributions but should be mentioned in the Conclusion and Discussion sections only.

**Response:**

We agree that limitations should not be presented in the contributions. We have therefore removed this statement from the bulleted list of contributions. The relevant limitations remain discussed in detail in the Discussion, particularly in the Strengths and Limitations subsection, and are also reflected in the Conclusion.

**Reviewer #1 – Comment 2**

The discussion from line number 398 to 414 of the revised manuscript gives the impression that the genuine scores vary between trial sessions. Hence, OCAV has low permanence. Permanence is a characteristic required for a human quality to be qualified as a biometric trait. Some justification for this should be included in the manuscript.

**Response:**

We agree that the significant difference in genuine score distributions between Visit 1 and Visits 2–3 requires a more detailed explanation in relation to short-term repeatability and permanence. The significant differences in genuine scores were found between Visit 1 and Visits 2–3, whereas no significant difference was observed between Visits 2 and 3. This finding does not indicate a continuous decline across the three sessions, but rather a shift associated primarily with the first visit. In addition, the violin plots in Fig. 13 show that the median genuine scores remained high and clearly separated from the impostor scores across all visits. The ROC curves in Fig. 12 similarly show that discrimination remained substantial, although the AUC decreased from 0.97 on Visit 1 to 0.94 and 0.92 on Visits 2 and 3, respectively. We have revised the Discussion to describe these findings.

**Reviewer #1 – Comment 3**

The authors should clarify that using participant-specific thresholds won’t affect the universality of the OCAV inversely.

**Response:**

We added clarification to the Discussion about the two thresholding strategies: global and participant-. In both strategies, the system is the same. The only difference is the decision threshold applied to the verification score. In a future implementation, a participant-specific threshold could be adjusted from each user’s enrollment samples. Such adjustment could accommodate inter-user score variability and would not restrict the range of users who might enroll in the system. Because this enrollment-based adjustment was not prospectively evaluated in the present study, the participant-specific results remain an upper-bound feasibility estimate.

**Reviewer #1 – Comment 4**

The given reason for the dismissal of PCA/autoencoders as “not applicable because manageable” is convincing. If there is a better reason, for example, interpretability or reproducibility, then there will be more confidence in the feature set chosen.

**Response:**

We revised the Feature extraction section to clarify that dimensionality reduction was not applied in order to preserve the direct interpretability of the predefined acoustic response and signal-quality descriptors.

**Reviewer #1 – Comment 5**

The manuscript should be checked for English language mistakes employing some human language expert or specialized language software in consent with the authors.

**Response:**

The manuscript was reviewed and corrected by a professional English-language reviewer.

We sincerely thank the Editor and Reviewers for their thoughtful and constructive feedback. We believe that the revisions address all the remaining points raised in the decision letter and have improved the clarity and precision of the manuscript. We hope that the revised manuscript is now suitable for publication in PLOS ONE.

We appreciate your time and consideration.

Sincerely,

Amit Kam
on behalf of all the authors
