# Supplementary material for: Biometric verification using oral acoustic reflections
Source: PLoS One. 2026 Sep 25;21(9):e0359160. doi: 10.1371/journal.pone.0359160 (PMC13614562; doi:10.1371/journal.pone.0359160)
Supplement: Peer review history

# Peer review: Decision Letter 0

**DOI:** 10.1371/journal.pone.0359160.r001

**Authors:** Amit Krishan Kumar

16 Jan 2026

Dear Dr. Kam,

Thank you for submitting your manuscript to PLOS ONE. After careful consideration, we feel that it has merit but does not fully meet PLOS ONE’s publication criteria as it currently stands. Therefore, we invite you to submit a revised version of the manuscript that addresses the points raised during the review process.

Please submit your revised manuscript by Mar 02 2026 11:59PM. If you will need more time than this to complete your revisions, please reply to this message or contact the journal office at plosone@plos.org. When you're ready to submit your revision, log on to https://www.editorialmanager.com/pone/ and select the 'Submissions Needing Revision' folder to locate your manuscript file.

- A letter that responds to each point raised by the academic editor and reviewer(s). You should upload this letter as a separate file labeled 'Response to Reviewers'.
- A marked-up copy of your manuscript that highlights changes made to the original version. You should upload this as a separate file labeled 'Revised Manuscript with Track Changes'.
- An unmarked version of your revised paper without tracked changes. You should upload this as a separate file labeled 'Manuscript'.

If you would like to make changes to your financial disclosure, please include your updated statement in your cover letter. Guidelines for resubmitting your figure files are available below the reviewer comments at the end of this letter.

If applicable, we recommend that you deposit your laboratory protocols in protocols.io to enhance the reproducibility of your results. Protocols.io assigns your protocol its own identifier (DOI) so that it can be cited independently in the future. For instructions see: https://journals.plos.org/plosone/s/submission-guidelines#loc-laboratory-protocols. Additionally, PLOS ONE offers an option for publishing peer-reviewed Lab Protocol articles, which describe protocols hosted on protocols.io. Read more information on sharing protocols at https://plos.org/protocols?utm_medium=editorial-email&utm_source=authorletters&utm_campaign=protocols.

We look forward to receiving your revised manuscript.

Kind regards,

Kumar, PhD

Academic Editor

PLOS One

Journal Requirements:

When submitting your revision, we need you to address these additional requirements.

1.Please ensure that your manuscript meets PLOS ONE's style requirements, including those for file naming. The PLOS ONE style templates can be found at

https://journals.plos.org/plosone/s/file?id=wjVg/PLOSOne_formatting_sample_main_body.pdf and

https://journals.plos.org/plosone/s/file?id=ba62/PLOSOne_formatting_sample_title_authors_affiliations.pdf

2. Please note that PLOS One has specific guidelines on code sharing for submissions in which author-generated code underpins the findings in the manuscript. In these cases, we expect all author-generated code to be made available without restrictions upon publication of the work. Please review our guidelines at https://journals.plos.org/plosone/s/materials-and-software-sharing#loc-sharing-code and ensure that your code is shared in a way that follows best practice and facilitates reproducibility and reuse.

3. Thank you for stating the following in the Competing Interests section:

“I have read the journal's policy and the authors of this manuscript have the following competing interests: Echo-ID Ltd. provided institutional support by funding the university’s conduct of this research. Amit Kam, Daniel Tubiana, Eran Hochstater and Roi Ram are employees of Echo-ID Ltd. This affiliation does not alter our adherence to PLOS ONE policies on sharing data and materials.”

We note that one or more of the authors are employed by a commercial company: name of commercial company.

1. Please provide an amended Funding Statement declaring this commercial affiliation, as well as a statement regarding the Role of Funders in your study. If the funding organization did not play a role in the study design, data collection and analysis, decision to publish, or preparation of the manuscript and only provided financial support in the form of authors' salaries and/or research materials, please review your statements relating to the author contributions, and ensure you have specifically and accurately indicated the role(s) that these authors had in your study. You can update author roles in the Author Contributions section of the online submission form.

Please also include the following statement within your amended Funding Statement.

“The funder provided support in the form of salaries for authors [insert relevant initials], but did not have any additional role in the study design, data collection and analysis, decision to publish, or preparation of the manuscript. The specific roles of these authors are articulated in the ‘author contributions’ section.”

If your commercial affiliation did play a role in your study, please state and explain this role within your updated Funding Statement.

2. Please also provide an updated Competing Interests Statement declaring this commercial affiliation along with any other relevant declarations relating to employment, consultancy, patents, products in development, or marketed products, etc.

Within your Competing Interests Statement, please confirm that this commercial affiliation does not alter your adherence to all PLOS ONE policies on sharing data and materials by including the following statement: "This does not alter our adherence to  PLOS ONE policies on sharing data and materials.” (as detailed online in our guide for authors http://journals.plos.org/plosone/s/competing-interests) . If this adherence statement is not accurate and  there are restrictions on sharing of data and/or materials, please state these. Please note that we cannot proceed with consideration of your article until this information has been declared.

Please include both an updated Funding Statement and Competing Interests Statement in your cover letter. We will change the online submission form on your behalf.

4. We note that you have indicated that there are restrictions to data sharing for this study. PLOS only allows data to be available upon request if there are legal or ethical restrictions on sharing data publicly. For more information on unacceptable data access restrictions, please see http://journals.plos.org/plosone/s/data-availability#loc-unacceptable-data-access-restrictions.

Before we proceed with your manuscript, please address the following prompts:

a) If there are ethical or legal restrictions on sharing a de-identified data set, please explain them in detail (e.g., data contain potentially identifying or sensitive patient information, data are owned by a third-party organization, etc.) and who has imposed them (e.g., a Research Ethics Committee or Institutional Review Board, etc.). Please also provide contact information for a data access committee, ethics committee, or other institutional body to which data requests may be sent.

b) If there are no restrictions, please upload the minimal anonymized data set necessary to replicate your study findings to a stable, public repository and provide us with the relevant URLs, DOIs, or accession numbers. For a list of recommended repositories, please see

https://journals.plos.org/plosone/s/recommended-repositories. You also have the option of uploading the data as Supporting Information files, but we would recommend depositing data directly to a data repository if possible.

We will update your Data Availability statement on your behalf to reflect the information you provide.

5. When completing the data availability statement of the submission form, you indicated that you will make your data available on acceptance. We strongly recommend all authors decide on a data sharing plan before acceptance, as the process can be lengthy and hold up publication timelines. Please note that, though access restrictions are acceptable now, your entire data will need to be made freely accessible if your manuscript is accepted for publication. This policy applies to all data except where public deposition would breach compliance with the protocol approved by your research ethics board. If you are unable to adhere to our open data policy, please kindly revise your statement to explain your reasoning and we will seek the editor's input on an exemption. Please be assured that, once you have provided your new statement, the assessment of your exemption will not hold up the peer review process.

6. We notice that your supplementary figures are uploaded with the file type 'Figure'. Please amend the file type to 'Supporting Information'. Please ensure that each Supporting Information file has a legend listed in the manuscript after the references list.

Additional Editor Comments:

The authors are encouraged to carefully consider and respond to all reviewer comments in a comprehensive and balanced manner to strengthen the overall quality of the manuscript.

Reviewer's Responses to Questions

**Comments to the Author**

1. Is the manuscript technically sound, and do the data support the conclusions?

Reviewer #1: Partly

Reviewer #2: Yes

**********

2. Has the statistical analysis been performed appropriately and rigorously?

Reviewer #1: No

Reviewer #2: No

**********

3. Have the authors made all data underlying the findings in their manuscript fully available?

The PLOS Data policy

Reviewer #1: Yes

Reviewer #2: Yes

**********

4. Is the manuscript presented in an intelligible fashion and written in standard English?

Reviewer #1: Yes

Reviewer #2: Yes

**********

Reviewer #1: • A biometric characteristic is related to a human physical or behavioral characteristic, while acoustic reflections are the response of the human body to some external factor.

• This research is based on an artificial setting in which sound waves are transmitted in the oral cavity, and the acoustic response is captured and characterized for verification purposes. It is evident that the raw data collection setup has limited acceptability among users and is also susceptible to serious hygiene issues.

• There should be debate among experts before employing this strategy as a biometric trait. According to the authors, this has never been used as a biometric trait in past research works.

• As there is no prior literature available for this biometric trait, the comparison of this trait with the existing physical and behavioral biometric traits, like finger-vein, face shape, gait, etc., is supposed to be part of the literature. So that the experts may decide whether oral acoustic qualifies as a biometric trait or not.

• To facilitate the experts, for the biometric trait claimed in this manuscript, the degree of the qualities a biometric trait has, like acceptability, universality, uniqueness, reliability, liveliness, hard to counterfeit, etc. are to be reported through some lab or social experiment for which data is collected from common people.

• If some international biometric standardization organization approves oral acoustic as a valid biometric trait, this manuscript may be accepted for publication after the standard review process.

• The specification for hardware resources and software employed is to be mentioned in detail. The computing machine capability and the libraries, toolboxes, and functions employed in experimental simulations are to be mentioned along with the parameters/ hyperparameters details for facilitating researchers interested in confirmation of the reported results and performing comparative analysis.

Reviewer #2: Manuscript is interesting and following points should be considered during revision.

1) Classical biometric traits are inherently linked to stable human physiological or behavioral characteristics. In contrast, acoustic reflections primarily represent the body’s response to externally injected stimuli, which raises questions about their classification as a true biometric attribute.

2) The proposed methodology relies on an artificial acquisition setup where sound waves are actively introduced into the oral cavity and the resulting acoustic response is analyzed for identity verification. Such a data acquisition process may face significant challenges in terms of user comfort, practical acceptability, and hygiene, particularly in real-world deployment scenarios.

3) Given that the authors claim novelty in introducing oral acoustic response as a biometric modality, a broader discussion within the biometric research community appears necessary before considering its adoption. To the best of the reviewer’s knowledge, this modality has not been formally established or validated as a biometric trait in prior studies.

4) The manuscript should provide comprehensive details of the experimental setup, including hardware specifications and software resources. Information regarding the computing platform, employed libraries or toolboxes, and all relevant parameters and hyperparameters used in simulations should be explicitly stated to ensure reproducibility and facilitate comparative studies by future researchers.

5) To support the proposed claim, an evaluation of standard biometric properties such as universality, acceptability, uniqueness, permanence, reliability, liveness detection, and resistance to spoofing should be conducted. Ideally, these properties should be quantified through controlled laboratory studies or socially representative experiments involving data collected from a diverse population.

6) The authors should consider referencing prior studies on respiratory and airway acoustic/impedance modeling, where anatomical and biomechanical properties of the oral–respiratory system were shown to produce distinctive and repeatable acoustic responses. In particular, respiratory-signal-based modeling approaches used for inhalation impedance estimation and respiratory pattern analysis could provide additional context supporting the uniqueness and stability of oral acoustic reflections used for biometric verification. I suggest authors cite this: 'Spirometer and sEMG respiratory patterns for clinical decision support system'. ( doi: 10.1109/I2MTC53148.2023.10175925 )

7) Since there is no established literature on oral acoustics as a biometric modality, the manuscript would benefit from a comparative discussion with well-known physical and behavioral biometrics (e.g., fingerprint, finger-vein, facial structure, gait). Such a comparison could help clarify whether oral acoustic characteristics meet the fundamental criteria required of a biometric trait.

8) Acceptance of oral acoustic response as a valid biometric modality would be significantly strengthened if it were endorsed or standardized by a recognized international biometric standardization body. Without such validation, the contribution remains preliminary.

**********

what does this mean?). If published, this will include your full peer review and any attached files.

If you choose “no”, your identity will remain anonymous but your review may still be made public.

**Do you want your identity to be public for this peer review?**  For information about this choice, including consent withdrawal, please see our Privacy Policy

Reviewer #1: No

Reviewer #2: No

**********

[NOTE: If reviewer comments were submitted as an attachment file, they will be attached to this email and accessible via the submission site. Please log into your account, locate the manuscript record, and check for the action link "View Attachments". If this link does not appear, there are no attachment files.]

To ensure your figures meet our technical requirements, please review our figure guidelines: https://journals.plos.org/plosone/s/figures

You may also use PLOS’s free figure tool, NAAS, to help you prepare publication quality figures: https://journals.plos.org/plosone/s/figures#loc-tools-for-figure-preparation.

NAAS will assess whether your figures meet our technical requirements by comparing each figure against our figure specifications.

Attachment Submitted filename: CommentsToAuthors.docx

# Author comment: Author response to Decision Letter 1

**DOI:** 10.1371/journal.pone.0359160.r002

2 Mar 2026

see response to reviewers docx file uploaded

Attachment Submitted filename: Response to reviewers.docx

# Peer review: Decision Letter 1

**DOI:** 10.1371/journal.pone.0359160.r003

**Authors:** Diego Forero

5 May 2026

Dear Dr. Kam,

Please submit your revised manuscript by Jun 19 2026 11:59PM. If you will need more time than this to complete your revisions, please reply to this message or contact the journal office at plosone@plos.org. When you're ready to submit your revision, log on to https://www.editorialmanager.com/pone/ and select the 'Submissions Needing Revision' folder to locate your manuscript file.

- A letter that responds to each point raised by the academic editor and reviewer(s). You should upload this letter as a separate file labeled 'Response to Reviewers'.
- A marked-up copy of your manuscript that highlights changes made to the original version. You should upload this as a separate file labeled 'Revised Manuscript with Track Changes'.
- An unmarked version of your revised paper without tracked changes. You should upload this as a separate file labeled 'Manuscript'.

If you would like to make changes to your financial disclosure, please include your updated statement in your cover letter. Guidelines for resubmitting your figure files are available below the reviewer comments at the end of this letter.

If applicable, we recommend that you deposit your laboratory protocols in protocols.io to enhance the reproducibility of your results. Protocols.io assigns your protocol its own identifier (DOI) so that it can be cited independently in the future. For instructions see: https://journals.plos.org/plosone/s/submission-guidelines#loc-laboratory-protocols. Additionally, PLOS ONE offers an option for publishing peer-reviewed Lab Protocol articles, which describe protocols hosted on protocols.io. Read more information on sharing protocols at https://plos.org/protocols?utm_medium=editorial-email&utm_source=authorletters&utm_campaign=protocols.

As the corresponding author, your ORCID iD is verified in the submission system and will appear in the published article. PLOS supports the use of ORCID, and we encourage all coauthors to register for an ORCID iD and use it as well. Please encourage your coauthors to verify their ORCID iD within the submission system before final acceptance, as unverified ORCID iDs will not appear in the published article. *Only*  the individual author can complete the verification step; PLOS staff *cannot*  verify ORCID iDs on behalf of authors.

We look forward to receiving your revised manuscript.

Kind regards,

Diego A. Forero, MD; PhD

Academic Editor

PLOS One

**Journal Requirements** :

If the reviewer comments include a recommendation to cite specific previously published works, please review and evaluate these publications to determine whether they are relevant and should be cited. There is no requirement to cite these works unless the editor has indicated otherwise.

**Editorial Office Comments** :

Please disregard the reports of Reviewer #2. As discussed with the Academic Editor, there is no need to respond to this reviewer.

[Note: HTML markup is below. Please do not edit.]

Reviewers' comments:

Reviewer's Responses to Questions

**Comments to the Author**

Reviewer #1: (No Response)

Reviewer #2: (No Response)

Reviewer #3: (No Response)

**********

2. Is the manuscript technically sound, and do the data support the conclusions?

Reviewer #1: Partly

Reviewer #2: No

Reviewer #3: Yes

**********

3. Has the statistical analysis been performed appropriately and rigorously?

Reviewer #1: N/A

Reviewer #2: No

Reviewer #3: No

**********

4. Have the authors made all data underlying the findings in their manuscript fully available?

The PLOS Data policy

Reviewer #1: No

Reviewer #2: No

Reviewer #3: Yes

**********

5. Is the manuscript presented in an intelligible fashion and written in standard English?

Reviewer #1: Yes

Reviewer #2: No

Reviewer #3: Yes

**********

Reviewer #1: The authors present a novel biometric verification system based on oral cavity acoustics (OCAV).

The authors have largely ignored the first-round comment regarding approval of this biometric trait by an international biometric standardization organization, and then they should proceed with the review process.

However, these observations are to be communicated to the authors.

• The abstract highlights novelty but remains largely descriptive. Please sharpen the novelty claim by explicitly contrasting OCAV with ear-canal and voice biometrics in terms of anatomical stability, spoofing resilience, and deployment feasibility.

• The authors have combined the Introduction section with the related literature section. It is advised to separate both sections. A tabular summary will enhance the readability of the related literature section, listing the methodology employed, main performance metrics reported, and the shortcomings.

• The claimed contributions of the research work are to be mentioned at the end of the related literature section in bulleted form.

• However, the authors have mentioned that sterilization has been performed periodically to handle hygiene issues. The main concern is still there, that is, the serious hygiene issue. As the authors have mentioned, the OCAV profile is generated and recorded when an authorized / unauthorized user inhales using the OCAV system-installed inhaler. Therefore, in real-life environments, there are severe hygiene issues.

• As there will be separate devices used during the capturing of the subject’s OCAV signature at the time of enrollment (in the lab) and at the time of verification/identification (noisy real-life environment). There are chances of variations in the captured OCAV signatures at the enrollment and verification stages. This issue is to be discussed, and an explanation is required to handle it.

• The authors have mentioned that during the pre-training session, the participating subjects were trained regarding the proper usage of the inhaler. Hence, the margin of error and rejection rate for genuine users are expected to be higher when implemented in real life. Further, this uniform inhalation requirement results in limited acceptability, universality, and reliability.

• The approval letter (approval no. 467-3) is required to be presented in the Appendix.

• The 342-dimensional feature vector is described but not justified. Please explain why this dimensionality was chosen and whether dimensionality reduction (e.g., PCA, autoencoders) was considered to improve efficiency.

• The function of the Isolation Forest model requires explanation when employed to label the outlier feature vectors.

• The LightGBM classifier is trained on an in-house dataset. Clarify whether cross-validation was used and discuss risks of overfitting given the relatively small participant pool. This will reassure readers about methodological rigor.

• The authors have reported both global and participant-specific thresholds. Please expand the discussion of implications for deployment, particularly the trade-off between generalizability and personalization, and scalability concerns.

• These figures have the following concerns:

o Fig. 2 (D) schematic diagram: needs clearer labeling of components and signal flow.

o Fig. 5 transfer functions: there is only one transmitted and reflected signal, while the caption states ‘Transfer functions of two different participants’. No transfer function h(t) is defined in this figure in some mathematical equation form. This figure is to be revised.

o ROC and violin plots (Figs. 10–17): captions and text should explain what the differences mean, not just present curves.

• The discussion underplays limitations. Please add explicit mention of inhalation variability, spoofing risks (e.g., artificial oral cavities), and hygiene challenges, along with preliminary mitigation strategies.

• The conclusion section must justify the claimed contributions by referring to the presented experimental results in the form of performance metrics or ROC graphs.

• The conclusion could be enriched by suggesting next steps: testing in noisy environments, integration into commercial devices, and longitudinal studies to assess stability over months/years. This would give the paper a stronger translational impact.

Reviewer #2: 1) Wen authors say "Biometric verification methods, such as face and fingerprint verification, are commonly used in smartphones, security systems, and medical devices." This statement is generic background knowledge and contributes almost no technical information. The mention of medical devices is vague; biometric authentication is not yet widely standardized in clinical devices, so this claim should be clarified or supported. No connection is established with acoustic biometrics or physiological acoustics, which is the actual topic.

2) Anti-spoofing research in face and fingerprint recognition is highly mature. Go get you research right first before making any claims.

3) Readers mainly look at abstract to know authors' intelligent level. “Current systems are not readily adaptable to biometric verification in oral substance-exchange devices such as inhalers, breathalyzers, and nicotine devices.” where is proof of this statement. It ignores existing voice biometrics, which already operate through the oral cavity. There is major motivational gap.

4) Missing details include:

a)Acoustic signal properties: frequency range, waveform type (chirp, sweep, impulse), sampling rate

b) Hardware configuration: microphone placement, speaker location

c) Feature extraction: spectral features?, resonant modes?, transfer function estimation?

d) Classifier: thresholding, machine learning model, template matching

e) Noise handling: saliva, breathing, tongue motion

f) Repeatability issues: mouth opening variability

Include ALL above details. Without it the work is useless.

5) Dataset description is minimal: gender distribution missing, ethnic diversity missing, dental conditions not reported,

orthodontics / dentures ignored

Oral cavity acoustics are heavily affected by: tongue position, saliva volume, jaw angle, dental structure

Report ALL above to make this article atleast useful

6) Give thorough information on trials. a) What defines a trial?; b) How are impostor trials generated?

7) Are impostors cross-subject comparisons or simulated attacks? Give a paragraph on this

8) Give MATHEMATICALLY and in TEXTS how session variability analyzed?

9) Show cross validation and ablation of it. One paragraph.

10) A thorough discussion and MATHEMATICAL MODEL of how environmental conditions were controlled?

11) Biometric systems should report: False Acceptance Rate (FAR), False Rejection Rate (FRR), Equal Error Rate (EER),

ROC or DET curves

12) There is No confidence intervals, No baseline comparisons, No statistical significance, No latency or computation cost shown.

13) Table 1 is scientifically wrong.

14) Have the courtesy to acknowledge the use of AI tools. Extremely large portions of the text show uniform phrasing patterns and stylistic structures that are commonly associated with generative AI-assisted writing. However, the manuscript does not contain any disclosure regarding the use of such tools. The authors are encouraged to clarify this aspect and include a formal statement on AI-assisted manuscript preparation. This also raises concerns on originality of the research.

15) “Potential applications include health monitoring with inhalers, alcohol testing with breathalyzers, and enforcement of nicotine-use restrictions.” This claims are speculative

16) “These capabilities can support public health and safety efforts, for example limiting youth access to nicotine products and monitoring impaired driving.” This is policy speculation rather than scientific contribution. It also introduces ethical and regulatory implications without discussing: privacy concerns, biometric storage security, spoofing resistance.

17) The work relies heavily on generic statements, provides minimal methodological detail and includes speculative application claims.

18) The authors’ response does not adequately satisfy the journal’s requirements regarding code availability and reproducibility.

While the authors state that they will release the trained LightGBM model, feature matrices, labels, and evaluation scripts, they explicitly acknowledge that the proprietary feature-extraction development framework and original model training procedure will not be shared. This omission is critical because the feature extraction pipeline constitutes the core methodological contribution of the work. Without access to the feature generation process, the community cannot reproduce the model inputs from the raw acoustic recordings.

Providing a trained model and precomputed feature matrices does not constitute reproducibility. At best, it allows partial verification of reported metrics under fixed inputs. It does not allow independent reconstruction of the analysis pipeline, nor does it allow verification that the reported features genuinely originate from the raw acoustic signals described in the manuscript.

In biometric verification systems, the feature extraction stage typically defines the discriminative representation of the biometric signal. Therefore, withholding the feature extraction framework effectively prevents: a) Independent validation of the acoustic signal processing pipeline, b) Reproduction of the 242-dimensional feature representation, c) Verification that the features correspond to physiologically meaningful acoustic characteristics, d) Re-training or stress-testing of the method under different experimental conditions

The statement that “retraining is not required to reproduce the published results” is not sufficient. Reproducibility requires that an independent researcher can regenerate the features, retrain the model, and reproduce the reported results starting from the raw data. The current proposal does not enable this.

Furthermore, the justification that the framework is proprietary does not override the journal’s reproducibility requirements. If the core algorithm cannot be released, the authors must either a) Provide the complete feature extraction code and training pipeline necessary to reproduce the results from raw recordings, or b) Provide a detailed, implementable description of the feature extraction process sufficient for independent reconstruction.

Without one of these options, the manuscript fails to meet the journal’s standards for transparency and reproducibility.

Unless the authors provide access to the full feature extraction pipeline and model training procedure, the manuscript does not comply with the journal’s code-sharing policy. In its current form, the study cannot be independently reproduced and therefore should not proceed to publication.

19) The Data Availability Statement should be revised to ensure that all primary data, including the raw acoustic recordings, are publicly accessible without restriction at the time of publication. If this cannot be done, the manuscript does not satisfy the journal’s data availability requirements.

20) The authors indicate that only the derived 242-dimensional feature matrix, trained LightGBM model, and evaluation scripts will be publicly deposited, while the raw acoustic recordings will only be available upon request. This is problematic for several reasons.

First, the raw recordings constitute the primary experimental data from which all results are derived. Restricting access to these data prevents independent verification of the signal processing steps and makes it impossible to confirm that the released feature matrix accurately reflects the underlying recordings.

Second, the claim that the raw recordings are “not required to reproduce the published results” is not acceptable. Reproducibility requires that independent researchers can reconstruct the entire analytical pipeline starting from the original data, not merely reproduce metrics from precomputed features supplied by the authors.

Third, providing data only “upon reasonable request” introduces a gatekeeping mechanism that conflicts with open-data principles and the journal’s requirement that materials necessary to replicate the findings be made publicly available without restriction.

Unless there are clear and justified ethical or legal constraints—which have not been demonstrated here—the raw de-identified recordings should be deposited in the same public repository as the other materials

21) All figures representation are pathetic. The fonts size, font type do not match. Most of it looks copied from other sources. Moreover, the authors' do not provide caption, maybe because they do not know what the diagram represents, because if they had overlooked, then 1 or 2 figures will have captions missing. In this case all figures are without captions. The quality of figures are bad and seem like snapshot from other sources. This is evident through varying parameters in figures. Authors also used white arrows. Authors chut make figures better.

22) The current experiments rely on a laboratory prototype and controlled acquisition conditions, which do not replicate the operational environment of the proposed applications (e.g., inhalers, breathalyzers, or nicotine devices). In such real-world settings, factors such as airflow, saliva presence, device geometry, user posture, and hygiene constraints can significantly alter the acoustic response of the oral cavity. Simply stating that the sensing unit could be embedded in personal oral devices does not resolve these concerns, as no experiments or simulations are presented to demonstrate robustness under such conditions. The manuscript should therefore acknowledge this limitation explicitly and avoid implying practical readiness for these applications without supporting experimental validation.

23) The study does not present empirical evaluation of universality, long-term permanence, liveness robustness, resistance to spoofing, or user acceptability, all of which are central criteria in determining whether a physiological signal can realistically function as a biometric trait. Simply discussing these criteria in the Discussion section does not substitute for experimental evidence.

24) The claim that oral acoustic response has not previously been used as a biometric identifier, a more rigorous comparative positioning against established biometric modalities is necessary. Without such evaluation, it is difficult to assess whether the proposed signal genuinely satisfies the foundational requirements expected of biometric traits.

25) At minimum, the manuscript should clearly frame the work as a preliminary feasibility study, avoid implying readiness as a biometric modality, and explicitly acknowledge that the majority of biometric validity criteria remain untested.

26) For proper reproducibility and comparative analysis, the manuscript should explicitly report:

a) Hardware specifications (CPU/GPU model, RAM, operating system)

b) Exact software versions (Python, libraries, toolkits)

c) Complete model configuration, including all LightGBM and Isolation Forest hyperparameters

d) Training configuration (random seeds, data splits, number of iterations, stopping criteria)

e) Any preprocessing or normalization steps applied before feature input

Without these details, it remains difficult for other researchers to replicate the reported results or perform meaningful comparisons. The Methods section should therefore provide a fully specified experimental configuration rather than a general description of the environment.

26) Show feature extraction block diagram clearly with all details.

27) Provide algorithm and pseudocode of the approach

28) The proposed method treats the acoustic signal as a generic feature vector for a gradient boosting classifier, which is an oversimplification of the underlying physical process. The oral cavity should be modeled explicitly as an acoustic system. The authors should implement acoustic transfer function identification, estimating the impulse or frequency response between the transmitted signal and the received response. In addition, the acoustic field within the oral cavity should be analyzed through modal decomposition based on the Helmholtz equation, allowing the extraction of resonance modes that can serve as physically meaningful biometric signatures.

29) The current approach lacks a physically interpretable model linking the measured reflections to the geometry of the oral cavity. The problem should be formulated as an inverse acoustic scattering or acoustic tomography problem, where reflected signals are used to infer structural properties of the cavity. Furthermore, the authors should incorporate classical system identification techniques (e.g., ARMA or state-space acoustic modeling) to estimate stable dynamic representations of the oral cavity response rather than relying solely on engineered features.

30) The machine learning framework remains relatively shallow and does not incorporate the known physics governing acoustic propagation. The authors should consider physics-informed neural networks (PINNs) so that the learned representations satisfy the governing acoustic equations (e.g., Helmholtz constraints). Additionally, the mapping between the transmitted excitation and the measured response should be modeled using neural operator frameworks (such as Fourier Neural Operators or DeepONet), which are specifically designed to learn functional mappings between signals in physical systems.

31) The verification process is currently formulated as a standard classifier, which is not aligned with modern biometric verification paradigms. The authors should adopt contrastive or metric-learning approaches (e.g., triplet loss or InfoNCE) to learn identity embeddings from acoustic responses. In addition, the decision stage should be formulated within a Bayesian verification framework, where likelihood ratios are used to evaluate the hypotheses of genuine versus impostor identity, which is standard practice in rigorous biometric system design.

32) The feature representation strategy remains purely statistical and does not explore structural properties of the acoustic response. The resonance spectra and response patterns should be analyzed using topological data analysis (e.g., persistent homology) to extract invariant descriptors of resonance structure. Furthermore, if cavity structure or response geometry can be inferred, the authors should explore geometric or graph-based learning methods, which are more appropriate for representing complex anatomical or acoustic manifolds.

33) While the study is described as a feasibility investigation, the manuscript still proposes a new biometric modality, which requires at least preliminary empirical assessment of the fundamental biometric properties beyond distinctiveness and short-term reliability.

The current evaluation remains limited to a relatively small and homogeneous cohort under controlled laboratory conditions. Key biometric characteristics—such as universality across broader populations, permanence over longer time scales, liveness detection, and resistance to spoofing—are not experimentally examined, yet these properties are central to determining whether a physiological signal can function as a practical biometric trait.

Simply stating that these evaluations are outside the scope of the present study is not sufficient, particularly when the manuscript discusses potential deployment in security-sensitive applications. At minimum, the manuscript should clearly limit its claims to a preliminary feasibility demonstration, and explicitly acknowledge that the majority of standard biometric validation criteria remain untested and require dedicated investigation before the modality can be considered viable.

34) Authors used very old references in their manuscript, include at least 20 more references from year 2022 to 2026.

35) Why is Omer Gross removed from authorship if the personal did the major work. The acknowledgement raises a concern regarding authorship attribution. The authors state that Omer Gross “provided support in conducting the experiments” and that her “assistance in data collection was essential to the successful completion of the experimental phase.” This description suggests a substantial contribution to the experimental work and primary data acquisition, which typically qualifies as a significant research contribution.

According to widely accepted authorship guidelines (e.g., ICMJE and similar editorial standards), individuals who make substantial contributions to the acquisition of data or experimental execution should normally be considered for authorship, particularly when their role is described as essential to the completion of the study.

The authors should therefore clarify the exact nature and extent of Omer Gross’s contribution. If she indeed played a central role in conducting the experiments and collecting the primary dataset used in the study, her contribution appears to meet the threshold for authorship rather than acknowledgement.

The authors should either: a) Include Omer Gross as a co-author, with her contributions clearly stated, or b) Provide a clear explanation confirming that the contribution does not meet the journal’s authorship criteria and that she has agreed to be acknowledged instead.

Clarification is necessary to ensure compliance with proper authorship and contribution standards.

36) The conclusion overstates the practical readiness and comparative performance of the proposed system. The study was conducted under controlled laboratory conditions with a limited cohort, yet the text implies performance comparable to established biometric systems and readiness for deployment in real devices. Moreover, an EER of approximately 12.5% is relatively high for biometric verification, and the claim of strong resistance to spoofing is not supported by dedicated adversarial experiments. The conclusion should therefore be moderated to reflect that the study represents a preliminary feasibility evaluation, and avoid claims of comparability with standard biometrics or real-world applicability without further large-scale, longitudinal, and security-focused validation.

Reviewer #3: The authors have done a commendable job addressing several of the concerns raised in the previous review round. However, before the manuscript can be fully accepted for publication, there are critical methodological clarifications regarding the machine learning pipeline and the physical constraints of the hardware that must be explicitly addressed in the text.

1. Critical Methodological Ambiguity

In the "Classification Model" section, the authors state: "We trained the LightGBM classifier on a pre-existing in-house dataset of 20,000 comparison vectors...". It is imperative to explicitly clarify the demographic origin of this "in-house dataset." Specifically, the authors must state whether the subjects in this training dataset are entirely mutually exclusive from the 108 subjects used in the testing cohort. If there is any subject overlap between the pre-existing training set and the test set, this would introduce a substantial risk of data leakage and could materially inflate the reported AUC and accuracy estimates. Please add a sentence or paragraph explicitly defining the separation between the training and testing sets to assure the readers of the model's generalizability and statistical rigor.

2. Fluid Dynamics and Inhalation Variance

The system relies on a pressure drop during inhalation to trigger the acoustic chirp. Under controlled laboratory conditions, subjects were trained to breathe uniformly. However, in real-world applications (e.g., impaired individuals using breathalyzers, or patients with varying respiratory capacities), inhalation force and velocity will fluctuate significantly. Such variations could affect the measured acoustic transfer function and therefore the stability of verification performance in real-world use.

Did the training or testing protocols evaluate intra-subject variance across different inhalation intensities? If this variable was controlled or assumed constant, it must be explicitly acknowledged as a constraint in the "Strengths and limitations" section, as natural respiratory variance could affect the stability of the transfer function in field deployments.

3. Environmental Degradation of the Acoustic Channel

In the limitations section, the authors appropriately mention the mechanical wear of the 3D-printed mouthpieces. However, for a device handling oral substance-exchange, acoustic sensors are highly susceptible to micro-environmental changes.

Please add a brief acknowledgment in the limitations section regarding the potential impact of moisture condensation or saliva accumulation within the acoustic channel over time. These factors can alter the resonance and dampen the acoustic reflections, which would be important to consider for longitudinal reliability outside the laboratory.

**********

what does this mean?). If published, this will include your full peer review and any attached files.

If you choose “no”, your identity will remain anonymous but your review may still be made public.

**Do you want your identity to be public for this peer review?**  For information about this choice, including consent withdrawal, please see our Privacy Policy

Reviewer #1: No

Reviewer #2: No

Reviewer #3: **Yes:**  Fabian León-Vargas

**********

[NOTE: If reviewer comments were submitted as an attachment file, they will be attached to this email and accessible via the submission site. Please log into your account, locate the manuscript record, and check for the action link "View Attachments". If this link does not appear, there are no attachment files.]

To ensure your figures meet our technical requirements, please review our figure guidelines: https://journals.plos.org/plosone/s/figures

You may also use PLOS’s free figure tool, NAAS, to help you prepare publication quality figures: https://journals.plos.org/plosone/s/figures#loc-tools-for-figure-preparation.

NAAS will assess whether your figures meet our technical requirements by comparing each figure against our figure specifications.

Attachment Submitted filename: CommentsToAuthors.docx

# Author comment: Author response to Decision Letter 2

**DOI:** 10.1371/journal.pone.0359160.r004

18 Jun 2026

All response to editor and reviewers are in the Response to Reviewers doc

Attachment Submitted filename: Response_to_Reviewers_auresp_2.docx

# Peer review: Decision Letter 2

**DOI:** 10.1371/journal.pone.0359160.r005

**Authors:** Diego Forero

7 Jul 2026

Dear Dr. Kam,

Please submit your revised manuscript by Aug 21 2026 11:59PM. If you will need more time than this to complete your revisions, please reply to this message or contact the journal office at plosone@plos.org. When you're ready to submit your revision, log on to https://www.editorialmanager.com/pone/ and select the 'Submissions Needing Revision' folder to locate your manuscript file.

- A letter that responds to each point raised by the academic editor and reviewer(s). You should upload this letter as a separate file labeled 'Response to Reviewers'.
- A marked-up copy of your manuscript that highlights changes made to the original version. You should upload this as a separate file labeled 'Revised Manuscript with Track Changes'.
- An unmarked version of your revised paper without tracked changes. You should upload this as a separate file labeled 'Manuscript'.

If you would like to make changes to your financial disclosure, please include your updated statement in your cover letter. Guidelines for resubmitting your figure files are available below the reviewer comments at the end of this letter.

If applicable, we recommend that you deposit your laboratory protocols in protocols.io to enhance the reproducibility of your results. Protocols.io assigns your protocol its own identifier (DOI) so that it can be cited independently in the future. For instructions see: https://journals.plos.org/plosone/s/submission-guidelines#loc-laboratory-protocols. Additionally, PLOS ONE offers an option for publishing peer-reviewed Lab Protocol articles, which describe protocols hosted on protocols.io. Read more information on sharing protocols at https://plos.org/protocols?utm_medium=editorial-email&utm_source=authorletters&utm_campaign=protocols.

As the corresponding author, your ORCID iD is verified in the submission system and will appear in the published article. PLOS supports the use of ORCID, and we encourage all coauthors to register for an ORCID iD and use it as well. Please encourage your coauthors to verify their ORCID iD within the submission system before final acceptance, as unverified ORCID iDs will not appear in the published article. *Only*  the individual author can complete the verification step; PLOS staff *cannot*  verify ORCID iDs on behalf of authors.

We look forward to receiving your revised manuscript.

Kind regards,

Diego A. Forero, MD; PhD

Academic Editor

PLOS One

Journal Requirements:

If the reviewer comments include a recommendation to cite specific previously published works, please review and evaluate these publications to determine whether they are relevant and should be cited. There is no requirement to cite these works unless the editor has indicated otherwise.

Please review your reference list to ensure that it is complete and correct. If you have cited papers that have been retracted, please include the rationale for doing so in the manuscript text, or remove these references and replace them with relevant current references. Any changes to the reference list should be mentioned in the rebuttal letter that accompanies your revised manuscript. If you need to cite a retracted article, indicate the article’s retracted status in the References list and also include a citation and full reference for the retraction notice.

[Note: HTML markup is below. Please do not edit.]

Reviewers' comments:

Reviewer's Responses to Questions

**Comments to the Author**

Reviewer #1: All comments have been addressed

Reviewer #3: All comments have been addressed

**********

2. Is the manuscript technically sound, and do the data support the conclusions?

Reviewer #1: Yes

Reviewer #3: Yes

**********

3. Has the statistical analysis been performed appropriately and rigorously?

Reviewer #1: Yes

Reviewer #3: Yes

**********

4. Have the authors made all data underlying the findings in their manuscript fully available?

The PLOS Data policy

Reviewer #1: No

Reviewer #3: Yes

**********

5. Is the manuscript presented in an intelligible fashion and written in standard English?

Reviewer #1: Yes

Reviewer #3: Yes

**********

Reviewer #1: Comments to Authors:

In response to the previous round comments, the authors have made significant changes. These observations are made from the revised manuscript:

• The Authors stated that the study was a controlled feasibility study rather than a standardized or fully validated biometric modality.

• Enhancements have been made to the abstract and introduction, which include contrasts between OCAV and existing biometric approaches, and a comparative literature table.

• Bulleted contributions, ethics approval paperwork, and better labeling of figures have been added.

• Increased discussion of limitations (hygiene issues, inhalation variation, spoofing risk, device variation) has been added.

• Technical details have been explained, including the 342-dimensional feature vector, Isolation Forest role, LightGBM training/testing separating and thresholding strategies.

• The claimed contributions have been linked to the derived conclusions and set future directions.

These points of improvement are to be communicated to the authors.

1. The authors have included this sentence in the main contributions (Identify current limitations related to controlled acquisition, inhalation variability, device consistency, hygiene, and the need for future validation). This sentence should not be written in the main contributions but should be mentioned in the conclusion and discussion sections only.

2. The discussion from line number 398 to 414 of the revised manuscript gives the impression that the genuine scores vary between trial sessions. Hence, OCAV has low permanence. Permanence is a characteristic required for a human quality to be qualified as a biometric trait. Some justification for this should be included in the manuscript.

3. The authors should clarify that using participant-specific thresholds won’t affect the universality of the OCAV inversely.

4. The given reason for the dismissal of PCA/autoencoders as “not applicable because manageable” is convincing. If there is a better reason, for example, interpretability or reproducibility, then there will be more confidence in the feature set chosen.

5. The manuscript should be checked for English language mistakes employing some human language expert or specialized language software in consent with the authors.

Reviewer #3: Comments were properly addresed. The OCAV system represents an innovative approach to biometric verification, and this foundational study provides a solid and honest framework for future longitudinal and real-world validations.

I recommend the manuscript for acceptance in its current form.

**********

what does this mean?). If published, this will include your full peer review and any attached files.

If you choose “no”, your identity will remain anonymous but your review may still be made public.

**Do you want your identity to be public for this peer review?**  For information about this choice, including consent withdrawal, please see our Privacy Policy

Reviewer #1: No

Reviewer #3: **Yes:**  Fabian León-Vargas

**********

[NOTE: If reviewer comments were submitted as an attachment file, they will be attached to this email and accessible via the submission site. Please log into your account, locate the manuscript record, and check for the action link "View Attachments". If this link does not appear, there are no attachment files.]

To ensure your figures meet our technical requirements, please review our figure guidelines: https://journals.plos.org/plosone/s/figures

You may also use PLOS’s free figure tool, NAAS, to help you prepare publication quality figures: https://journals.plos.org/plosone/s/figures#loc-tools-for-figure-preparation.

NAAS will assess whether your figures meet our technical requirements by comparing each figure against our figure specifications.

Attachment Submitted filename: CommentsToAuthors.docx

# Author comment: Author response to Decision Letter 3

**DOI:** 10.1371/journal.pone.0359160.r006

19 Aug 2026

our response is in the response letter

Attachment Submitted filename: Response_to_Reviewers_auresp_3.docx

# Peer review: Decision Letter 3

**DOI:** 10.1371/journal.pone.0359160.r007

**Authors:** Diego Forero

10 Sep 2026

Biometric verification using oral acoustic reflections

PONE-D-25-49343R3

Dear Dr. Kam,

We’re pleased to inform you that your manuscript has been judged scientifically suitable for publication and will be formally accepted for publication once it meets all outstanding technical requirements.

Within one week, you’ll receive an e-mail detailing the required amendments. When these have been addressed, you’ll receive a formal acceptance letter and your manuscript will be scheduled for publication.

An invoice will be generated when your article is formally accepted. Please note, if your institution has a publishing partnership with PLOS and your article meets the relevant criteria, all or part of your publication costs will be covered. Please make sure your user information is up-to-date by logging into Editorial Manager at Editorial Manager® and clicking the ‘Update My Information' link at the top of the page. For questions related to billing, please contact billing support.

If your institution or institutions have a press office, please notify them about your upcoming paper to help maximize its impact. If they’ll be preparing press materials, please inform our press team as soon as possible -- no later than 48 hours after receiving the formal acceptance. Your manuscript will remain under strict press embargo until 2 pm Eastern Time on the date of publication. For more information, please contact onepress@plos.org.

Kind regards,

Diego A. Forero, MD; PhD

Academic Editor

PLOS One

Additional Editor Comments (optional):

Reviewers' comments:

Reviewer's Responses to Questions

**Comments to the Author**

Reviewer #1: All comments have been addressed

**********

2. Is the manuscript technically sound, and do the data support the conclusions?

Reviewer #1: Yes

**********

3. Has the statistical analysis been performed appropriately and rigorously?

Reviewer #1: Yes

**********

4. Have the authors made all data underlying the findings in their manuscript fully available?

The PLOS Data policy

Reviewer #1: Yes

**********

5. Is the manuscript presented in an intelligible fashion and written in standard English?

Reviewer #1: Yes

**********

Reviewer #1: In response to the previous round comments, the authors have made significant changes. All the communicated observations have been addressed in the revised manuscript.

However, I still feel that it is required to perform longer span longitudinal study to evaluate the degree of OCAV permanence and other biometric characteristics. This point has already been acknowledged by the authors at line number 421 of the revised manuscript.

The manuscript has also been reviewed by human English language expert. Therefore, in my opinion, the manuscript may be accepted in the current form.

**********

what does this mean?). If published, this will include your full peer review and any attached files.

If you choose “no”, your identity will remain anonymous but your review may still be made public.

**Do you want your identity to be public for this peer review?**  For information about this choice, including consent withdrawal, please see our Privacy Policy

Reviewer #1: No

**********

Attachment Submitted filename: CommentsToAuthors.docx

# Editor report: Acceptance letter

**DOI:** 10.1371/journal.pone.0359160.r008

**Authors:** Diego Forero

PONE-D-25-49343R3

PLOS One

Dear Dr. Kam,

I'm pleased to inform you that your manuscript has been deemed suitable for publication in PLOS One. Congratulations! Your manuscript is now being handed over to our production team.

At this stage, our production department will prepare your paper for publication. This includes ensuring the following:

* All references, tables, and figures are properly cited

* All relevant supporting information is included in the manuscript submission,

* There are no issues that prevent the paper from being properly typeset

You will receive further instructions from the production team, including instructions on how to review your proof when it is ready. Please keep in mind that we are working through a large volume of accepted articles, so please give us a few days to review your paper and let you know the next and final steps.

Lastly, if your institution or institutions have a press office, please let them know about your upcoming paper now to help maximize its impact. If they'll be preparing press materials, please inform our press team within the next 48 hours. Your manuscript will remain under strict press embargo until 2 pm Eastern Time on the date of publication. For more information, please contact onepress@plos.org.

You will receive an invoice from PLOS for your publication fee after your manuscript has reached the completed accept phase. If you receive an email requesting payment before acceptance or for any other service, this may be a phishing scheme. Learn how to identify phishing emails and protect your accounts at https://explore.plos.org/phishing.

If we can help with anything else, please email us at customercare@plos.org.

Thank you for submitting your work to PLOS One and supporting open access.

Kind regards,

PLOS One Editorial Office Staff

on behalf of

Dr. Diego A. Forero

Academic Editor

PLOS One
